# Evaluating the halogen bonding strength of a iodoloisoxazolium(III) salt

**DOI:** 10.3762/bjoc.20.204

**Published:** 2024-09-23

**Authors:** Dominik L Reinhard, Anna Schmidt, Marc Sons, Julian Wolf, Elric Engelage, Stefan M Huber

**Affiliations:** 1 Fakultät für Chemie und Biochemie, Ruhr-Universität Bochum, Universitätsstraße 150, 44801 Bochum, Germany

**Keywords:** diaryliodonium, gold catalysis, halogen bonding, hypervalent iodine, non-covalent interactions

## Abstract

Diaryliodonium(III) salts have been established as powerful halogen-bond donors in recent years. Herein, a new structural motif for this compound class was developed: iodoloisoxazolium salts, bearing a cyclic five-membered iodolium core fused with an isoxazole ring. A derivative of this class was synthesized and investigated in the solid state by X-ray crystallography. Finally, the potential as halogen-bonding activator was benchmarked in solution in the gold-catalyzed cyclization of a propargyl amide.

## Introduction

**Figure 1:**
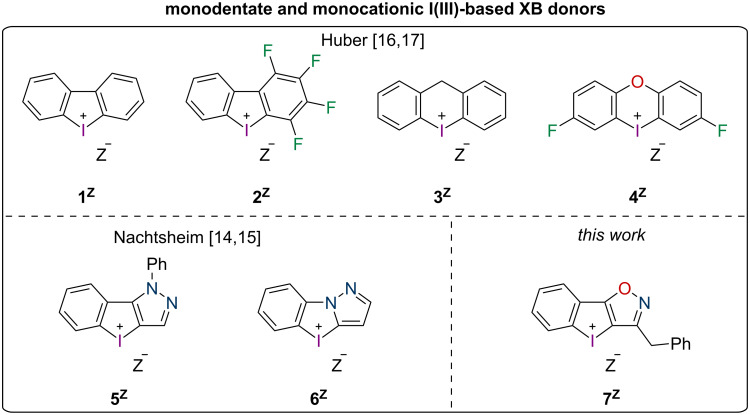
Set of literature-known monocationic cyclic diaryliodonium(III) salts that were applied as XB donors (Z = OTf, BArF_24_).

The compound class of diaryliodonium (DAI) salts has been known since the end of the 19th century and their use as aryl-transfer reagents has been widely explored [1–3]. The application as Lewis acid catalysts, on the other hand, has only gained interest in the last ten years after a first report by Han and Liu in 2015 on their use as catalysts in a Mannich reaction [4]. In 2018, our group showed in a proof-of-principle study [5] that the Lewis acid catalysis by DAI salts is based on halogen bonding (XB), an interaction between a Lewis base (XB acceptor) and an electrophilic halogen atom in the Lewis acid (XB donor) [6–10]. In organocatalysis, previously only iodine(I)-based Lewis acids had been applied. However, after this study, the application of DAI salts as XB donors gained increasing interest and was investigated by several groups [11]. In the last years, important information about structure–activity relationships was also obtained: in a titration study by Mayer and Legault it was determined that cyclic five-membered DAI salts, so-called iodolium compounds, are significantly stronger Lewis acids than their less-stable acyclic counterparts [12]. By using the activation of alkyl halides as a benchmark, our group later reported that six-membered core structures are also weaker XB donors (iodininium **3^OTf^**) than iodolium **1^OTf^** [13]. Furthermore, the importance of substituents in the core and on the outer rings was demonstrated (XB donors **2^OTf^** and **4^OTf^**). Nachtsheim reported the synthesis of *N*-heterocyclic substituted monocationic iodolium salts like derivatives **5^Z^** and **6^Z^** (Figure 1) [14–15]. Their benchmark studies showed significant activity differences amongst them and superior performance compared to prototypical iodolium **1^Z^**. Significant upgrades to DAI-based XB catalysts were made in the form of bidentate and dicationic XB donors [16–17] from our group as well as of dicationic *N*-heterocyclic-substituted monodentate catalysts by Nachtsheim [15]. While such compounds are necessary to activate neutral substrates in more challenging reactions, monodentate and monocationic congeners provide sufficient activation in halide abstractions, e.g. to activate gold chloride complexes [18–19]. Therefore, besides the development of new bidentate catalyst motifs, we were still interested in the optimization of these “simpler” derivatives. Thus, we designed a new catalyst motif [20] featuring an isoxazole ring, XB donor **7^Z^**, and compared it with our known iodonium species in the activation of Au(I)–Cl bonds.

## Results and Discussion

**Scheme 1:**
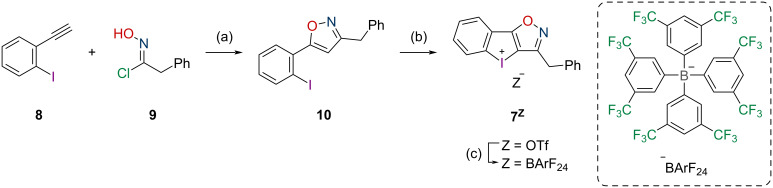
Synthesis of the iodoloisoxazolium salts **7^Z^**: (a) 1.5 equiv **9**, 0.2 equiv CuI, 2.0 equiv K_2_CO_3_, (THF), 0.1 M, 135 h at rt, 43%, (b) 1.5 equiv mCPBA, 3.0 equiv TfOH (at 0 °C), (DCM), 0.1 M, 20 h at rt, 85%, (c) 1.0 equiv NaBArF_24_, (acetone), 0.5 M, 2 h at 50 °C under microwave irradiation, 72%.

As immediate precursor to the target structure **7^Z^**, the literature-known isoxazole **10** was synthesized via a Cu(I)-catalyzed cycloaddition between (2-iodophenyl)acetylene (**8**) and benzyl nitrile oxide, which is produced in situ from the imidoyl chloride **9** [21]. The one-pot oxidation and ring-closure reaction [22–23] to iodoloisoxazolium(III) salt **7^OTf^** and the salt metathesis with sodium tetrakis(3,5-bis(trifluoromethyl)phenyl)borate (NaBArF_24_) were then realized with 85% and 72% yield, respectively (Scheme 1).

**Figure 2:**
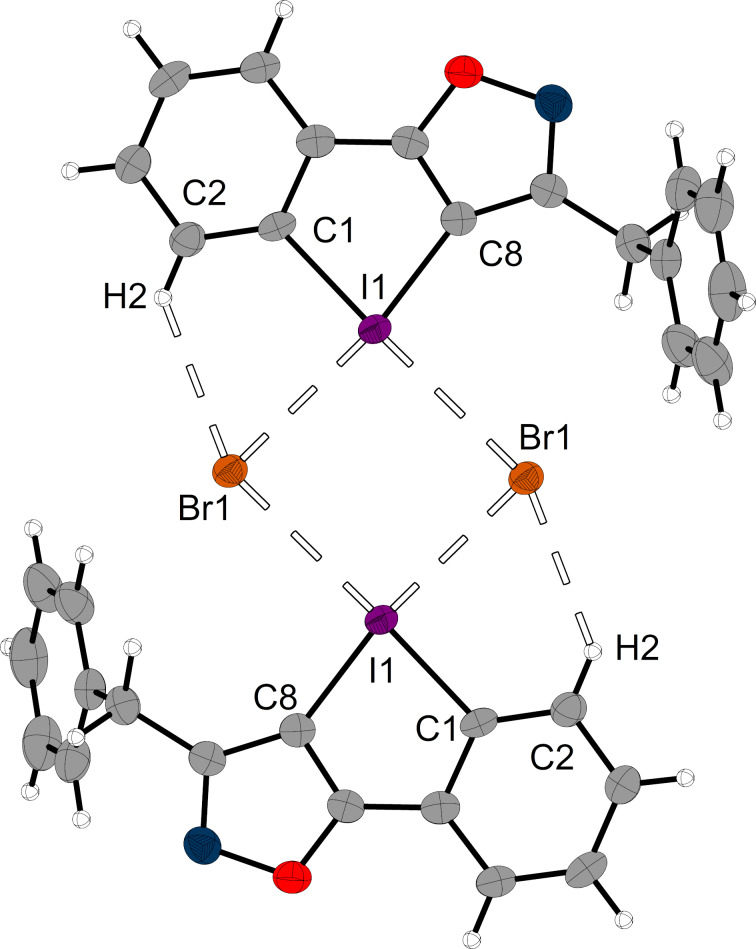
Halogen bonding dimer found in the crystal structure of **7^Br^**. Ellipsoids are shown at 50% probability (carbon: grey, nitrogen: blue, oxygen: red, bromine: orange, iodine: purple) and hydrogen atoms are shown in standard ball-and-stick model (white). Halogen and hydrogen bonding is indicated dashed.

The triflate salt **7^OTf^** was transformed into the corresponding bromide salt by XB-activated solvolysis of α-methylbenzyl bromide in wet acetonitrile [13]. The DAI salt **7^Br^** crystallized from this solution in the monoclinic space group *P*2_1_/*n* with a cell volume of 1447.91(3) Å^3^ (*a* = 5.4707(1) Å, *b* = 10.9139(1) Å, *c* = 24.3668(3) Å, β = 95.601(1)°) and a density of 2.01865 g/cm^3^. Two units of the cationic XB donor form a dimer, which is bridged via two bromide ions (Figure 2). As usual for DAI salts, two XB axes are found on the elongations of the C–I bonds. On the one *trans* to the isoxazolium unit, halogen bonding [I1···Br1 = 3.0610(5) Å, 80% of Σr, and C8–I1···Br1 = 171.67(9)°] and hydrogen bonding were found [H2···Br1 = 2.7991(4) Å, 95% of Σr, C2···Br1 = 3.545(4) Å, 100% of Σr and C2–H2···Br1 = 136.1(2)°]. On the other axis, no ortho proton is present, so only XB is observed [I1···Br1 = 3.2023(5) Å, 84% of Σr, and C1–I1···Br1 = 176.08(9)°]. The bond distances indicate that the hydrogen bond is noticeably weaker than the two XBs and thus constitutes merely an assisting interaction.

The XB interactions in this crystal structure were compared to the ones in the literature-known co-crystal of prototypic iodolium **1^BArF^** with bromide (CCDC: 1145291) [5]. For the latter, such a dimeric binding motif was also found, with I–Br bond lengths of 3.1936(9) Å [83% of Σr] and 3.2299(9) Å [84% of Σr]. It can be concluded that stronger halogen bonding can be found in the crystal structure of iodoloisoxazolium **7^Br^**, which hints that also in solution stronger binding to Lewis bases and therefore higher activity as catalyst may be expected (compared to prototypic iodolium **1^Z^**).

**Scheme 2:**
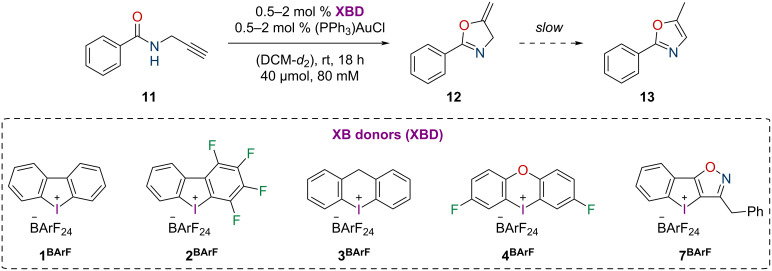
Gold(I)-catalyzed cyclization of propargylic amide **11** as benchmark reaction for Au–Cl activation.

As a benchmark for the halogen-bonding strength in solution, the activation of (PPh_3_)AuCl was chosen. The activated gold(I) complex was applied as catalyst for the cyclization of propargylic amide **11**, a typical benchmark reaction in gold catalysis (Scheme 2) [24–27], which had previously already been activated by iodine(I) and iodine(III)-based XB donors [15,18].

To evaluate the activity of the new iodoloisoxazolium **7^BArF^**, it was compared to the four monodentate iodine(III)-based XB donors **1^BArF^**–**4^BArF^** (Scheme 2), which had been applied in a previous study by our group as triflate salts and which had shown strong differences in XB donor strength [13]. While the six-membered iodininium salt **3^OTf^** proved to be markedly weaker than prototypic iodolium **1^OTf^**, the oxygen-bridged iodoxinium **4^OTf^** exhibited improved performance and the polyfluorinated iodolium **2^OTf^** was by far the most active. A previous study on gold activation by halogen bonding showed significantly higher activity when the weakly coordinating counteranion tetrakis[3,5-bis(trifluoromethyl)phenyl]borate (^−^BArF_24_) was used instead of triflate [18]. Therefore, standard anion metathesis procedures were employed to prepare the salts **1^BArF^**–**4^BArF^** (see Supporting Information File 1).

**Figure 3:**
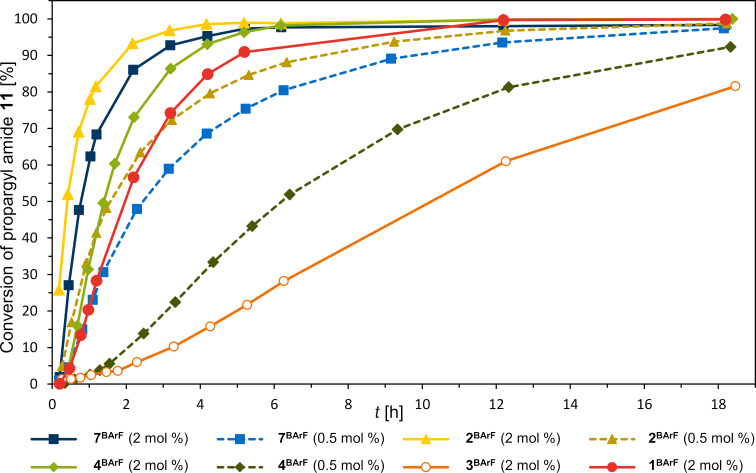
^1^H NMR kinetics of the gold-catalyzed cyclization shown in Scheme 2. An equimolar amount of the gold complex was applied, respectively. Every experiment was performed three times (see Supporting Information File 1).

Similarly to our previous report on this gold activation, the gold complex (PPh_3_)AuCl was applied with a catalyst loading of 2 mol %, activated by an equal amount of the DAI salt. Due to solubility issues, the reaction had to be performed in methylene chloride instead of chloroform. The gold-catalyzed cyclization reaction (Scheme 2) was followed via ^1^H NMR spectroscopy (Figure 3).

When applying the six-membered cyclic DAI salt **3^BArF^** as an activator, the lowest activity was observed, reaching ≈80% conversion after 18 h (≈5% after 2 h). It has to be noted that a sigmoidal curve was observed. This indicates that the activity of the catalyst system increases over time. A preactivation process between the XB donor, the gold complex, or the amide can be assumed. Such a sigmoidal curve for this reaction has also been observed in one of the previous studies on the XB activation of gold complexes [15]. The prototypic iodolium **1^BArF^** showed significantly better results reaching ≈85% conversion after already 4 h (≈55% after 2 h) and the oxygen-bridged iodoxinium **4^BArF^** performed slightly better (≈70% after 2 h). For these two catalysts, a very slight sigmoidal curve shape was also observed. The polyfluorinated XB donor **2^BArF^** performed the best, with ≈90% conversion after 2 h. The resulting order of catalytic activity of these halogen-bond donors is in line with the above-mentioned previous benchmark of these activators [13]. Finally, also the new iodoloisoxazolium salt **7^BArF^** was applied and a comparably high activity was observed (ca. 85% conversion after 2 h). This result marks this halogen-bond donor as the second-best activator out of this set of compounds. Furthermore, the three strongest XB donors **2^BArF^**, **4^BArF^**, and **7^BArF^** were also applied at a catalyst loading of 0.5 mol % (with an equimolar amount of (PPh_3_)AuCl). Both, the tetrafluoroiodolium **2^BArF^** and the iodoloisoxazolium **7^BArF^** are still effective activators even at such low catalyst-system loadings. The tetrafluoroiodolium **2^BArF^** yields a conversion of almost 80% and the iodoloisoxazolium **7^BArF^** one of almost 70% after 4 h. In comparison, the iodoxinium salt **4^BArF^** also featured a sigmoidal curve shape and a significantly slower activation, which results in an amide consumption of only ca. 30% after 4 hours. To quantify the activity differences of these XB donors, the reaction kinetics were fitted according to a pseudo-first-order rate. Only selected periods (the first four data points within the first 1.5 hours of reaction) were considered, as such a coarse approximation cannot be applied after reaching the plateau due to equilibrium processes (see Supporting Information File 1 for further details). The determined TOF of tetrafluoroiodolium salt **2^BArF^** reaches a value of 80 h^−1^, almost 1.7 times as high as the TOF of iodoloisoxazolium **7^BArF^** with 48 h^−1^. Both TOFs are much higher (almost 5 and 3 times higher) than the one of iodoxinium **4^BArF^** with 17 h^−1^ (Table 1).

**Table 1:** Determined TOFs of the strongest activators **2^BArF^**, **7^BArF^**, and **4^BArF^** (and their calculated standard deviation). The TOFs were determined from the kinetics (see Supporting Information File 1 for further details).

XB donor	TOF [h^−1^]

**2** **^BArF^**	80 ± 7
**7** **^BArF^**	48 ± 4
**4** **^BArF^**	17 ± 1

Since the three compounds **2^BArF^**–**4^BArF^** have not been tested in this reaction before, and iodoloisoxazolium salt **7^BArF^** has not been tested in any reaction at all, several control experiments were also performed, even though the benchmark reaction has already been established in halogen-bonding activation. In the presence of 2 mol % of either the unactivated gold complex (PPh_3_)AuCl or the XB donors **1^BArF^**–**4^BArF^** + **7^BArF^**, ^1^H NMR showed no conversion within 18 h, indicating that the activated gold complex is the catalytically active species. Furthermore, stability measurements (^1^H and ^19^F NMR) of 1:1 mixtures of the gold complex and the XB donors were performed in order to investigate the stability of the cationic iodonium structures towards the gold complex [28]. For all catalyst systems, decomposition of the ^−^BArF_24_ anion was observed via ^1^H and ^19^F NMR spectroscopy, which is known to happen in the presence of activated gold complexes [29]. The stability of the DAI cations was checked with ^1^H NMR: the characteristic doublets belonging to the respective iodonium structures **1^+^**, **2^+^**, **3^+^**, and **7^+^** were found to be constant (see Supporting Information File 1). The signals of the iodoxinium cation **4^+^** were overlapping with signals of the anion. However, the stability of **4^+^** (as well as of **2^+^**) could be confirmed by ^19^F NMR measurements: no decomposition of the signals belonging to the core structure of the cations was observed. These results indicate that the DAI cations are still intact and do not decompose in the presence of the gold complex. In previous works, the mode of activation by several XB donors including DAI salts was investigated, suggesting that halide abstraction is the crucial step towards the formation of a catalytically active gold species [18–19]. Furthermore, iodonium species **1^BArF^**–**4^BArF^** have been shown to be halide abstracting agents in the Ritter-type solvolysis of α-methylbenzyl bromide and via the crystal structures of **1^Cl^**, **2^Cl^**, and **3^Cl^** which resulted from crystallization of the respective cation with the abstracted chloride from the Ritter-type solvolysis of benzhydryl chloride [13]. The crystal structure of **5^Br^** was also obtained directly from the halide-abstraction reaction (see Supporting Information File 1). These three facts and the considerations mentioned before, strongly hint that the same kind of halide abstraction from the gold(I) species is occurring here with the presented XB donors **1^BArF^**–**4^BArF^** and **7^BArF^**.

## Conclusion

In this study, we reported the synthesis of a new cyclic diaryliodonium motif: the iodoloisoxazolium unit bearing a five-membered iodolium core fused with an isoxazole ring. The derivatives **7^Z^** (Z = OTf, BArF_24_) were synthesized and the crystal structure of the corresponding bromide salt was determined. Its analysis provided cases of strong halogen bonding, which was further investigated in solution via the activation of the gold–chlorine bond in the catalyst (PPh_3_)AuCl. Here, the new diaryliodonium motif outcompeted other XB donors like the prototypical iodolium **1^BArF^** and showed a similar activity as the polyfluorinated XB donor **2^BArF^**. The results illustrate the potential of the iodoloisoxazolium for halogen-bonding activation and catalysis. Studies on the synthesis and application of chiral and/or bidentate dicationic derivatives are currently underway in our laboratory.

## Supporting Information

File 1 Synthesis, catalyses, and characterization data.

File 2 Crystallographic information file of **7^Br^**.

File 3 Crystallographic data.

File 4 Crystallographic data.

## Data Availability

The data that supports the findings of this study is available from the corresponding author upon reasonable request.
