# Supplementary material for: Evaluating the halogen bonding strength of a iodoloisoxazolium(III) salt
Source: Beilstein J Org Chem. 2024 Sep 23;20:2401–7. doi: 10.3762/bjoc.20.204 (PMC11443664; doi:10.3762/bjoc.20.204)
Supplement: File 1 — Synthesis, catalyses, and characterization data. [file Beilstein_J_Org_Chem-20-2401-s001.pdf]

BEILSTEIN JOURNAL OF ORGANIC CHEMISTRY

Supporting Information

for

Evaluating the halogen bonding strength of a iodoloisoxazolium(III) salt

Dominik L. Reinhard, Anna Schmidt, Marc Sons, Julian Wolf, Elric Engelage and Stefan M. Huber

Beilstein J. Org. Chem. **2024,** *20,* 2401–2407. doi:10.3762/bjoc.20.204

Synthesis, catalyses, and characterization data

License and Terms: This is a supporting information file under the terms of the Creative Commons Attribution License (https://creativecommons.org/licenses/by/4.0). Please note that the reuse, redistribution and reproduction in particular requires that the author(s) and source are credited and that individual graphics may be subject to special legal provisions.
The license is subject to the *Beilstein Journal of Organic Chemistry* terms and conditions: (https://www.beilstein-journals.org/bjoc/terms)

Table of contents

1. General remarks S2
   1.1 Chemicals and solvents S2
   1.2 Methods for synthesis and chromatography S2
   1.3 Analytical methods S2
   1.4 Synthesis of known XB donors S2
2. Synthesis of unknown XB donors S3
   2.1 3-Benzyl-5-(2-iodophenyl)isoxazole (**13**) S3
   2.2 3-Benzylbenzo[4,5]iodolo[2,3-*d*]isoxazol-4-ium trifluoromethanesulfonate (**7**^{OTf}) S4
   2.3 3-Benzylbenzo[4,5]iodolo[2,3-*d*]isoxazol-4-ium tetrakis(3,5-bis(trifluoromethyl)phenyl)borate (**7**^{BArF}) S5
   2.4 1,2,3,4-Tetrafluorodibenzo[*b*,*d*]iodol-5-ium tetrakis(3,5-bis(trifluoromethyl)phenyl)borate (**2**^{BArF}) S6
   2.5 10*H*-Dibenzo[*b*,*e*]iodinin-5-ium tetrakis(3,5-bis(trifluoromethyl)phenyl)borate (3^{BArF}) S7
   2.6 3,7-Difluorodibenzo[*b*,*e*][1,4]iodaoxin-5-ium tetrakis(3,5-bis(trifluoromethyl)phenyl)borate (**4**^{BArF}) S8
3. Catalytic experiments S9
   3.1 Synthesis of the starting material S9
   3.2 General procedures for catalysis experiments S9
   3.3 Replica of performed catalytic experiments with 2 mol % catalyst system: S9
   3.4 Replica of performed catalytic experiments with 0.5 mol % catalyst system S14
   3.5 Determination of turnover-frequencies (TOFs) of **7**^{BArF} and **2**^{BArF} S16
   3.6 Control experiments S17
4. Crystallography S24
   4.1 Crystallization of **7**^{Br} S24
   4.2 Crystal structure data S24
5. Literature S29
6. Appendix – NMR spectra S30

1. General remarks

1.1 Chemicals and solvents

All used fine chemicals and solvents were purchased from commercial sources and were used without further purification, if not stated otherwise. If bought in technical grade pentane, ethyl acetate, dichloromethane and diethyl ether were used after single distillation. Dry dichloromethane, diethyl ether and tetrahydrofuran were received from an MBRAUN MB SPS-800. Other dry solvents were obtained by storage over flame-dried 3 Å or 4 Å molecular sieves under argon. The water content of solvents was determined with a Karl Fischer Titroline®7500KF trace from SI Analytics with Honeywell (Fluka) Hydranal Coulomat-AD. Deuterated dichloromethane for catalysis experiments was stored over activated 3 Å molecular sieves.

1.2 Methods for Synthesis and Chromatography

All moisture or air-sensitive reactions were performed using standard Schlenk technique under argon as inert gas using dry solvents and flame- or oven-dried glassware, if necessary. Thin layer chromatography was performed using Merck TLC aluminum sheets (silica gel 60, F254) and spots were visualized using a UV lamp at 254 nm. For column chromatography silica gel of grain size 0.04–0.063 mm (Machery-Nagel Si60) was used. It was performed under atmospheric or increased pressure (by use of a hand- or peristaltic pump). Used eluents and retardation factors (R_f) are mentioned in the experiments.

1.3 Analytical methods

NMR spectra were measured on a Bruker DPX 250, AVIII 300, AVIII 400, DRX400, or Neo 400 spectrometer at ca. 300 K. Chemical shifts (δ) are given in parts per million (ppm) and are internally referenced to tetramethylsilane by residual solvent signals.[1] Spectra were analyzed using MestReNova. Multiplicities are abbreviated as s (singlet), d (doublet), t (triplet), q (quartet), p (pentet), sept (septet), m (multiplet), and combinations of those, e.g. td (triplet of doublets). The relative integral and the coupling constant (J in Hz) are indicated if possible. IR spectra were obtained using a Shimadzu IR Affinity – 1S spectrometer with a Specac-Quest ATR unit. Peaks are reported in $\tilde{\upsilon} = cm^{-1}$ and are indicated with w (weak), m (medium), s (strong), vs (very strong) or br (broad). CHNS Elemental Analysis was performed with a vario Micro cube from Elementar Analysentechnik. Electron-spray ionization mass spectrometry (ESI–MS) was performed on a Bruker Daltonics Esquire 6000 or on an Advion Expression L compact instrument.

1.4 Synthesis of known XB donors

The literature-known XB donors $\mathbf{1}^{\mathbf{BArF}}$ [1], $\mathbf{2}^{\mathbf{OTf}}$ [2,3], $\mathbf{3}^{\mathbf{OTf}}$ [3], and $\mathbf{4}^{\mathbf{OTf}}$ [3] were prepared as described in the literature.

2. Synthesis of unknown XB donors

2.1 3-Benzyl-5-(2-iodophenyl)isoxazole (10)

The synthesis over three steps was performed adapting a procedure by Xu, Gao et al.[4]

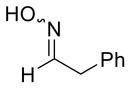

S1

Condensation to oxime: A solution of 837 mg Na_2CO_3 (CAS: 497-19-8, 7.9 mmol, 0.5 equiv) in 3 mL H_2O was added dropwise to a solution of 1.8 mL phenylacetaldehyde (CAS: 122--78-1, 1.85 g, 15.4 mmol, 1.0 equiv) and 1.21 g hydroxylammonium hydrochloride (CAS: 5470-11-1, 17.4 mmol, 1.1 equiv) in 50 mL of 50% aqueous EtOH (0.3 M). The reaction was subsequently stirred at rt for 3 h and after the addition of 25 mL water, the mixture was extracted thrice with EtOAc (50 mL each). The combined organic extracts were washed with water (50 mL) and saturated aqueous NaCl (50 mL), dried over $MgSO_4$, filtered, and the solvent was removed in vacuo yielding the crude oxime **S1** (CAS: 7028-48-0) as E/Z-mixture, which was directly subjected to the next step.

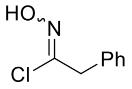

9

Chlorination of oxime: A solution of 2.31 g *N*-chlorosuccinimide (CAS: 128-09-6, 17.3 mmol, 1.1 equiv) in 15 mL DMF was added dropwise to a solution of the crude oxime **S1** (CAS: 7028-48-0, assuming 15.4 mmol, ≤ 1.0 equiv) in 15 mL DMF (total volume 1.0 M). The solution was stirred for 1 h at 40 °C bath temperature and after cooling to rt, water and EtOAc (75 mL each) were added. The phases were separated and the aqueous one was extracted twice with EtOAc (75 mL each). The combined organic extracts were washed with water and saturated aqueous NaCl (75 mL each). After drying over $MgSO_4$, the solvent was removed in vacuo (threefold co-evaporation of DMF with heptane) yielding the crude imidoyl chloride **9** (CAS: 701-72-4) as an E/Z mixture, which was directly subjected to the next step without purification.

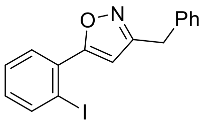

10

Copper-catalyzed cycloaddition: Prior to use, dry THF was degassed by 1 h of argon bubbling. In a brown glass Schlenk flask, the crude imidoyl chloride **9** (CAS: 701-72-4, assuming 15.4 mmol, ≤ 1.5 equiv) was dissolved in 100 mL dry and degassed THF (0.1 M corresponding to **8**). To this, 2.3 g of alkyne **8** (CAS: 766-50-7, 10 mmol, 1.0 equiv), 387 mg copper(I) iodide (CAS: 7681-65-4, 20 mol % Cu^I), and 2.81 g K_2CO_3 (CAS: 584-08-7, 2.0 equiv) were added in argon counterflow. After the suspension was stirred at rt for 135 h, it was filtered through a plug of celite with aid of Et_2O. The filtrate was washed three times with a basic EDTA solution (50 mL each), once with water, and once with saturated aqueous NaCl (50 mL each). After drying over $MgSO_4$, the solvent was removed in vacuo. The product was purified via silica column chromatography (silica loading, 20:1 hexanes:EtOAc, R_f = 0.24).

Yield: 1.56 g (4.32 mmol, 43%) of a brownish oil.

^{1}H NMR (400 MHz, $CDCl_3$): δ = 7.98 (dd, *J* = 8.0, 1.1 Hz, 1H), 7.66 (dd, *J* = 7.8, 1.7 Hz, 1H), 7.43 (td, *J* = 7.7, 1.2 Hz, 1H), 7.38 – 7.29 (m, 4H), 7.29 – 7.22 (m, 1H), 7.10 (td, *J* = 7.7, 1.7 Hz, 1H), 6.64 (s, 1H), 4.11 (s, 2H) ppm. The NMR data fits the literature.[4]

2.2 3-Benzylbenzo[4,5]iodolo[2,3-*d*]isoxazol-4-ium trifluoromethanesulfonate (7^{OTf})

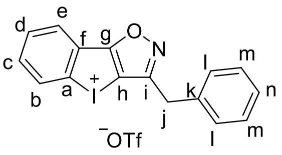

7^{OTf}

First, 137 mg of 3-benzyl-5-(2-iodophenyl)isoxazole (**10**, CAS: 2369671-89-4, 2.06 mmol, 1.0 equiv) were dissolved in 20 mL DCM (0.1 M). After the addition of 693 mg mCPBA (CAS: 937-14-4, ≤ 77%, ≤ 3.09 mmol, ≤ 1.5 equiv), the reaction mixture was cooled to 0 °C. Subsequently, 0.55 mL TfOH (CAS: 1493-13-6, 0.93 g, 6.2 mmol, 3.0 equiv) were added dropwise. After stirring for further 30 min at 0 °C, the cooling bath was removed, and the mixture was stirred at rt for 20 h. The solvent was removed *in vacuo* at rt using an HV pump and an external cold trap. Then, 40 mL Et_2O were added, and the suspension was stirred until homogenous. The precipitate was collected via vacuum filtration (Buchner funnel and paper filter), washed thrice with Et_2O, and dried in HV.

Yield: 892 mg (1.75 mmol, 85 %) of a yellowish solid.

^{1}H NMR (400 MHz, DMSO-*d_6*): δ = 8.38 (d, *J* = 8.4 Hz, 1H, C[b]-**H**), 8.30 (dd, *J* = 7.7, 1.5 Hz, 1H, C[e]-**H**), 7.92 (t, *J* = 7.5 Hz, 1H, C[d]-**H**), 7.80 (ddd, *J* = 8.8, 7.3, 1.6 Hz, 1H, , C[c]-**H**), 7.38 – 7.31 (m, 4H, C[l]-**H** & C[m]-**H**), 7.33 – 7.22 (m, 1H, C[n]-**H**), 4.39 (s, 2H, C[j]-**H_2**) ppm.

^{19}F NMR (235 MHz, DMSO-*d6*): δ = -77.72 (s, $^{-}O_3S$-C**F_3**) ppm.

^{13}C{^{1}H} NMR (75 MHz, DMSO-*d_6*): δ = 175.8 (**C**[g]), 160.8 (**C**[i]), 136.6 (**C**[k]), 132.7 (**C**[c]), 131.3 (**C**[b] or **C**[d]), 131.2 (**C**[b] or **C**[d]), 128.7 (**C**[m] or **C**[n]), 128.6 (**C**[m] or **C**[n]), 128.3 (**C**[f]), 126.8 (**C**[l]), 126.5 (**C**[e]), 125.3 (**C**[a]), 120.7 (q, *J* = 322.4 Hz, $^{-}O_3S$-**C**F_3), 93.1 (**C**[h]), 30.6 (**C**[j]) ppm.

ESI-MS: m/z (+) = calc. 359.99 $[M\text{-}OTf]^{+}$, found 359.77 $[M\text{-}OTf]^{+}$

m/z (-) = calc. 148.95 $[OTf]^{-}$, found 148.74 $[OTf]^{-}$

ATR-IR [$\tilde{\nu}$ = cm^{-1}]: 515 (s), 575 (m), 631 (s), 656 (m), 714 (m), 729 (s), 768 (m), 818 (w), 864 (w), 937 (w), 1018 (s), 1161 (m), 1173 (m), 1184 (m), 1215 (s), 1233 (m), 1288 (m), 1408 (m), 1433 (w), 1497 (w), 1541 (w), 1593 (w).

m.p.: decomposition > 190 °C

EA: calc.: 40.10 %C, 2.18 %H, 2.75 %N, 6.30 %S, found: 40.28 %C, 2.23 %H, 2.70 %N, 6.25 %S.

2.3 3-Benzylbenzo[4,5]iodolo[2,3-*d*]isoxazol-4-ium tetrakis(3,5-bis(trifluoromethyl)phenyl)borate (7^{BArF})

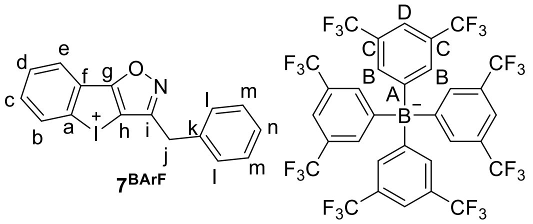

In a microwave vial, 511 mg iodoloisoxazolium triflate $\mathbf{7^{OTf}}$ (1.00 mmol, 1.0 equiv) and 889 mg $NaBArF_{24}$ (CAS: 79060-88-1, 1.00 mmol, 1.0 equiv) were mixed with 2.0 mL dry acetone (0.5 M). The vial was placed in a CEM Discover SP microwave reactor and the reaction was stirred in dynamic mode at 50 °C for 2 h under microwave irradiation. After cooling to rt, the mixture was filtered (paper filter) and washed thrice with 6 mL acetone. The solvent of the filtrate was removed in vacuo. The residue was suspended in 7 mL chloroform, filtered (paper filter), and washed thrice with 1 mL chloroform. To the filtrate, 40 mL *n*-pentane were added, and a yellow-orange oil separated. The mixture was shortly sonicated and then stored at 5 °C (fridge) for 30 min. The supernatant was afterward removed via a syringe. The oily residue was subsequently washed thrice with 6 mL *n*-pentane and dried in HV, yielding the product containing 0.8 equiv acetone (determined via ^{1}H NMR spectroscopy, mass purity of 96 %).

Yield: 915 mg (96 % mass purity, 718 µmol, 72 %) of an orange solid.

^{1}H NMR (400 MHz, DMSO-*d*$_6$): δ = 8.39 (d, *J* = 8.3 Hz, 1H, C[b]-**H**), 8.27 (dd, *J* = 7.7, 1.5 Hz, 1H, C[e]-**H**), 7.89 (td, *J* = 7.5, 1.0 Hz, 1H, C[d]-**H**), 7.78 (ddd, *J* = 8.7, 7.4, 1.5 Hz, 1H, C[c]-**H**), 7.62 (d, *J* = 3.9 Hz, 12H, C[B]-**H** & C[D]-**H**), 7.38 – 7.29 (m, 4H, C[l]-**H** & C[m]-**H**), 7.27 – 7.20 (m, 1H, C[n]-**H**), 4.40 (s, 2H, C[j]-**H$_2$**) ppm.

^{19}F NMR (376 MHz, DMSO-*d*$_6$): δ = -61.95 (C[C]-C**F$_3$**) ppm.

^{13}C{^{1}H} NMR (101 MHz, DMSO-*d*$_6$): δ = 175.8 (**C**[g], 161.0 (dd, *J* = 99.8, 49.6 Hz, **C**[A]), 160.7 (**C**[i]), 136.6, 134.1 (**C**[B]), 132.7 (**C**[c]), 131.3 (**C**[b] or **C**[d]), 131.2 (**C**[b] or **C**[d]), 128.7 (**C**[m] or **C**[l]), 128.6 (**C**[m] or **C**[l]), 129.0 – 127.9 (m, **C**[C]), 128.3 (**C**[f]), 126.7 (**C**[n]), 126.4 (**C**[e]), 125.3 (**C**[a]), 124.0 (q, *J* = 272.6 Hz, C[C]-**C**F$_3$), 118.1 – 116.8 (m, **C**[D]), 93.12 (**C**[h]), 30.6 (**C**[j], overlap with acetone) ppm.

ESI-MS: m/z (+) = calc. 359.99 [M-BArF$_{24}$]$^+$, found 360.2 [M-BArF$_{24}$]$^+$
m/z (-) = calc. 863.06 [BArF$_{24}$]$^-$, found 863.1 [BArF$_{24}$]$^-$

ATR-IR [$\tilde{\nu}$ = cm^{-1}]: 667 (s), 682 (s), 710 (m), 715 (m), 727 (m), 744 (w), 761 (m), 838 (m), 886 (m), 1072 (m), 1114 (s), 1158 (m), 1273 (s), 1352 (m), 1686 (w).

m.p.: 130 °C

EA: calc.: 47.67 %C, 2.21 %H, 1.10 %N, found: 48.28 %C, 2.05 %H, 1.04 %N.
(including 0.8 equiv acetone)

2.4 1,2,3,4-Tetrafluorodibenzo[*b*,*d*]iodol-5-ium tetrakis(3,5-bis(trifluoromethyl)phenyl)borate (2^{BArF})

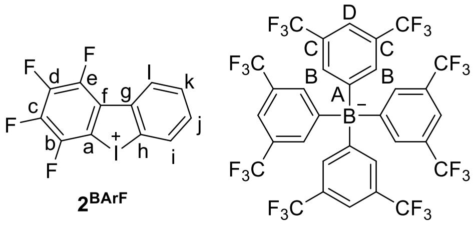

In a microwave vial, 251 mg iodolium triflate $\mathbf{2^{OTf}}$ (502 µmol, 1.0 equiv) and 444 mg $NaBArF_{24}$ (CAS: 79060-88-1, 501 µmol, 1.0 equiv) were dissolved in 1.5 mL MeOH (0.3 M). The vial was placed in a CEM Discover SP microwave reactor and the reaction was stirred in dynamic mode at 50 °C for 2 h under microwave irradiation. After cooling to rt, the solvent was removed in vacuo. The residue was suspended in 3 mL chloroform and filtered. The solvent of the filtrate was removed in vacuo. The residue was redissolved in a small amount of DCM and poured into *n*-pentane. An oil formed on the bottom, which solidified by storing the mixture at −30 °C. The solvent layer was decanted, and the residue was washed with *n*-pentane. The residue was co-evaporated three times with chloroform and dried for several days in HV.

Yield: 398 mg (328 µmol, 66 %) of a yellow foam.

^{1}H NMR (300 MHz, DMSO-*d*$_6$): δ = 8.40 (dd, *J* = 8.1, 1.5 Hz, 1H, C[l]-**H**), 8.36 (dd, *J* = 8.5, 1.1 Hz, 1H, C[i]-**H**), 7.92 (t, *J* = 7.9 Hz, 1H, C[k]-**H**), 7.80 (ddd, *J* = 8.7, 7.3, 1.5 Hz, 1H, C[j]-**H**), 7.62 (s, 12H, C[B]-**H** & C[D]-**H**) ppm.

^{19}F{^{1}H} NMR (235 MHz, DMSO-*d*$_6$): δ = -61.98 (s, 24F, C[C]-C**F$_3$**), -124.56 (ddd, *J* = 23.0, 11.2, 4.0 Hz, 1F, C[b]-**H** or C[e]-**H**), -139.02 (ddd, *J* = 20.3, 11.2, 4.7 Hz, 1F, C[b]-**H** or C[e]-**H**), -150.69 (td, *J* = 20.3, 4.0 Hz, 1F, C[c]-**H** or C[d]-**H**), -151.67 (ddd, *J* = 23.1, 20.2, 4.7 Hz, 1F, C[c]-**H** or C[d]-**H**) ppm.

^{13}C{^{1}H} NMR (101 MHz, DMSO-*d*$_6$): δ = 161.0 (dd, *J* = 99.6, 49.8 Hz, **C**[A]), 147.9 – 137.8 (m, **C**[b], **C**[c], **C**[d] & **C**[e]), 137.8 – 137.4 (m, **C**[g]), 134.1 (**C**[B]), 131.9 (**C**[j]), 131.6 (**C**[k]), 131.3 (**C**[i]), 130.0 (d, *J* = 16.1 Hz, (**C**[l]), 129.1 – 127.9 (m, **C**[C]), 127.2 – 126.8 (m, **C**[f]), 124.0 (q, *J* = 272.4 Hz, C[C]-**C**F$_3$), 121.8 (**C**[h]), 117.8 – 117.2 (m, **C**[D]), 103.6 (dt, *J* = 25.2, 2.8 Hz, **C**[a]) ppm.

ESI-MS: m/z (+) = calc. 350.93 $[M\text{-}BArF_{24}]^+$, found 350.69 $[M\text{-}BArF_{24}]^+$

m/z (-) = calc. 863.06 $[BArF_{24}]^-$, found 862.67 $[BArF_{24}]^-$

ATR-IR [$\tilde{\nu}$ = cm^{-1}]: 635 (m), 667 (s), 681 (s), 712 (s), 727 (w), 745 (m), 762 (m), 804 (m), 839 (m), 862 (w), 885 (m), 986 (m), 1030 (s), 1103 (vs), 1271 (vs), 1352 (s), 1427 (w), 1462 (w), 1497 (m), 1514 (m), 1611 (w).

m.p.: 122 °C

EA: calc.: 43.52 %C, 1.33 %H, found: 43.47 %C, 1.30 %H.

2.5 10*H*-Dibenzo[*b*,*e*]iodinin-5-ium tetrakis(3,5-bis(trifluoromethyl)phenyl)borate (3^{BArF})

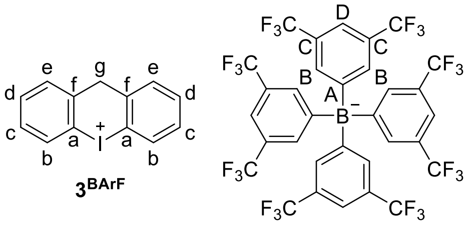

In a microwave vial, 221 mg iodinium triflate $\mathbf{3^{OTf}}$ (CAS: 1983120-62-2, 500 µmol, 1.0 equiv) and 444 mg $NaBArF_{24}$ (CAS: 79060-88-1, 501 µmol, 1.0 equiv) were dissolved in 1.5 mL MeOH (0.3 M). The vial was placed in a CEM Discover SP microwave reactor and the reaction was stirred in dynamic mode at 50 °C for 2 h under microwave irradiation. After cooling to rt, the solvent was removed in vacuo. The residue was suspended in 3 mL chloroform and filtered. The precipitate was suspended in 3 mL DCM and filtered. The filtrates of both steps were combined, and the solvents were removed in vacuo. The residue was redissolved in a small amount of DCM and precipitated with *n*-pentane. The solvent layer was decanted, and the residue was washed with *n*-pentane. The residue was co-evaporated thrice with chloroform and dried for several days in HV, yielding the product containing ≈0.9 equiv of chloroform (mass purity: 92%).

Yield: 462 mg (92 % mass purity, 368 µmol, 74 %) of a colorless solid.

^{1}H NMR (300 MHz, DMSO-*d*$_6$): δ = 8.11 (dd, *J* = 8.1, 1.2 Hz, 2H, C[b]-**H**), 7.79 (dd, *J* = 7.6, 1.6 Hz, 2H, C[e]-**H**), 7.68 – 7.60 (m, 12H, C[B]-**H** & C[D]-**H**), 7.57 (td, *J* = 7.5, 1.2 Hz, 2H, C[d]-**H**), 7.40 (td, *J* = 7.8, 1.7 Hz, 2H, C[c]-**H**), 4.32 (s, 2H, C[g]-$\mathbf{H_2}$) ppm.

^{19}F{^{1}H} NMR (235 MHz, DMSO-*d*$_6$): δ = -62.01 (C[C]-C$\mathbf{F_3}$) ppm.

^{13}C{^{1}H} NMR (75 MHz, DMSO-*d*$_6$): δ = 161.0 (dd, *J* = 99.6, 49.7 Hz, **C**[A]), 138.9 (**C**[f]), 134.1 (**C**[B]), 133.6 (**C**[b]), 131.7 (**C**[d]), 130.5 (**C**[e]), 129.0 (**C**[c]), 129.2 – 127.7 (m, **C**[C]), 124.0 (q, *J* = 272.4 Hz, C[B]-**C**F_3), 117.9 – 116.7 (m, **C**[D]), 116.0 (**C**[a]), 45.7 (**C**[g]) ppm.

ESI-MS: m/z (+) = calc. 292.98 $[M\text{-}BArF_{24}]^+$, found 292.72 $[M\text{-}BArF_{24}]^+$
m/z (-) = calc. 863.06 $[BArF_{24}]^-$, found 862.67 $[BArF_{24}]^-$

ATR-IR [ṽ = cm^{-1}]: 604 (m), 640 (m), 667 (s), 681 (s), 716 (s), 745 (s), 760 (m), 839 (m), 887 (s), 930 (w), 949 (w), 982 (m), 999 (m), 1105 (vs), 1111 (vs), 1159 (s), 1219 (w), 1271 (s), 1352 (s), 1443 (w), 1460 (w), 1611 (w).

m.p.: 148 °C

EA: calc.: 43.62 %C, 1.83 %H, found: 43.46 %C, 1.68 %H.
(including 0.9 equiv $CHCl_3$)

2.6 3,7-Difluorodibenzo[*b*,*e*][1,4]iodaoxin-5-ium tetrakis(3,5-bis(trifluoromethyl)phenyl)borate (4^{BArF})

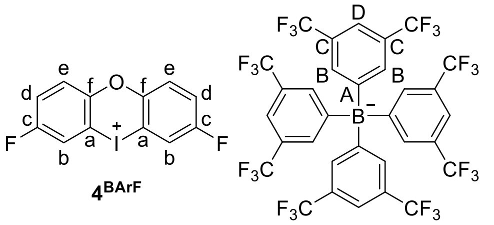

4BArF

In a microwave vial, 241 mg iodoxinium triflate $\mathbf{4}^{\mathbf{OTf}}$ (CAS: 2605749-34-4, 502 µmol, 1.0 equiv) and 445 mg $NaBArF_{24}$ (CAS: 79060-88-1, 502 µmol, 1.0 equiv) were dissolved in 1.5 mL MeOH (0.3 M). The vial was placed in a CEM Discover SP microwave reactor and the reaction was stirred in dynamic mode at 50 °C for 2 h under microwave irradiation. After cooling to rt, the solvent was removed in vacuo. The residue was suspended in 3 mL chloroform and filtered. The solvent of the filtrate was removed in vacuo. The residue was redissolved in a small amount of DCM and poured into *n*-pentane. An oil formed, which solidified by storing the mixture at −30 °C. The solvent layer was decanted, and the residue was washed with *n*-pentane. The residue was co-evaporated six times with chloroform and dried for several days in HV, yielding the product containing ≈0.3 equiv of chloroform and ≈0.3 equiv of DCM (mass purity: 95%).

Yield: 534 mg (95% mass purity, 425 µmol, 85%) of a beige solid.

^{1}H NMR (300 MHz, DMSO-*d*$_6$): δ = 7.89 (dd, *J* = 7.4, 3.0 Hz, 2H, C[b]-**H**), 7.73 (dd, *J* = 9.0, 4.6 Hz, 2H, C[e]-**H**), 7.68 – 7.58 (m, 12H, C[B]-**H** & C[D]-**H**), 7.52 (ddd, *J* = 9.1, 8.1, 3.0 Hz, 2H, C[d]-**H**) ppm.

^{19}F{^{1}H} NMR (235 MHz, DMSO-*d*$_6$): δ = -62.07 (s, 24F, C[C]-C**F**$_3$), -113.52 (s, 2F, C[c]-**F**) ppm.

^{13}C{^{1}H} NMR (75 MHz, DMSO-*d*$_6$): δ = 161.1 (dd, *J* = 99.6, 49.8 Hz, **C**[A]), 159.3 (d, *J* = 248.3 Hz, **C**[c]), 150.5 (d, *J* = 2.8 Hz, **C**[f]), 134.1 (**C**[B]), 129.3 – 127.6 (m, **C**[C]), 124.0 (q, *J* = 272.4 Hz, C[C]-**C**F$_3$), 122.6 (d, *J* = 8.6 Hz, **C**[e]), 120.4 (d, *J* = 23.7 Hz, **C**[d]), 120.0 (d, *J* = 27.9 Hz, **C**[b]), 117.7 – 117.1 (m, **C**[D]), 103.3 (d, *J* = 9.7 Hz, **C**[a]) ppm.

ESI-MS: m/z (+) = calc. 330.94 $[M\text{-}BArF_{24}]^+$, found 330.66 $[M\text{-}BArF_{24}]^+$
m/z (-) = calc. 863.06 $[BArF_{24}]^-$, found 862.67 $[BArF_{24}]^-$

ATR-IR [ῦ = cm^{-1}]: 586 (w), 667 (s), 681 (s), 716 (m), 746 (m), 768 (w), 799 (m), 826 (w), 839 (m), 856 (m), 887 (m), 932 (w), 951 (w), 1115 (vs), 1159 (s), 1273 (vs), 1352 (s), 1466 (m), 1491 (w), 1578 (w), 1593 (w), 1611 (w).

m.p.: 139 °´C

EA: calc.: 42.66 %C, 1.52 %H, found: 42.68 %C, 1.41 %H.
(including 0.3 equiv $CHCl_3$ + 0.3 equiv CH_2Cl_2)

3. Catalytic experiments

3.1 Synthesis of the starting material

Propargylic amide **11** was synthesized according to literature procedures [5].

3.2 General procedures for catalysis experiments

a) <u>2 mol % catalyst loading</u>

An NMR tube was evacuated and refilled with argon. The stock solutions were added under ambient atmosphere. The stock solutions (in DCM-d_2) were added in the following order: propargylic amide **11** (300 µL; 133 mM; 40 µmol; 1 equiv, including tetraethylsilane [33 mM, 10 µmol, 0.25 equiv] as an internal standard), XB Donor (100 µL; 8 mM; 0.8 µmol; 0.02 equiv), chloro(triphenylphosphine) gold (100 µL; 8 mM; 0.8 µmol; 0.02 equiv). The tube was closed, inverted at least three times and then measured periodically on a Bruker AVIII 300 MHz device.

b) <u>0.5 mol % catalyst loading</u>

An NMR tube was evacuated and refilled with argon. The stock solutions were added under ambient atmosphere. The stock solutions (in DCM-d_2) were added in the following order: propargylic amide **11** (400 µL; 100 mM; 40 µmol; 1 equiv, including tetraethylsilane [44 mM, 13 µmol, 0.33 equiv] as an internal standard) or 100 µL DCM-d_2 and propargylic amide **11** (300 µL; 133 mM; 40 µmol; 1 equiv, including tetraethylsilane [33 mM, 10 µmol, 0.25 equiv] as an internal standard), XB Donor (50 µL; 4 mM; 0.2 µmol; 0.0005 equiv), chloro(triphenylphosphine)gold (50 µL; 4 mM; 0.2 µmol; 0.0005 equiv). The tube was closed, inverted at least three times and then measured periodically on a Bruker AVIII 300 MHz device.

3.3 Replica of performed catalytic experiments with 2 mol % catalyst system:

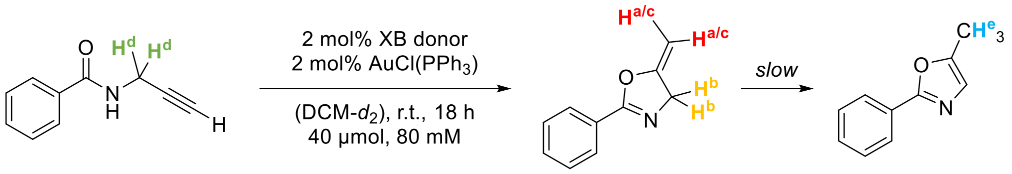

11 12 13

Scheme S1: Schematic representation of the activation of propargylic amide **11** with a catalyst system loading of 2 mol %.

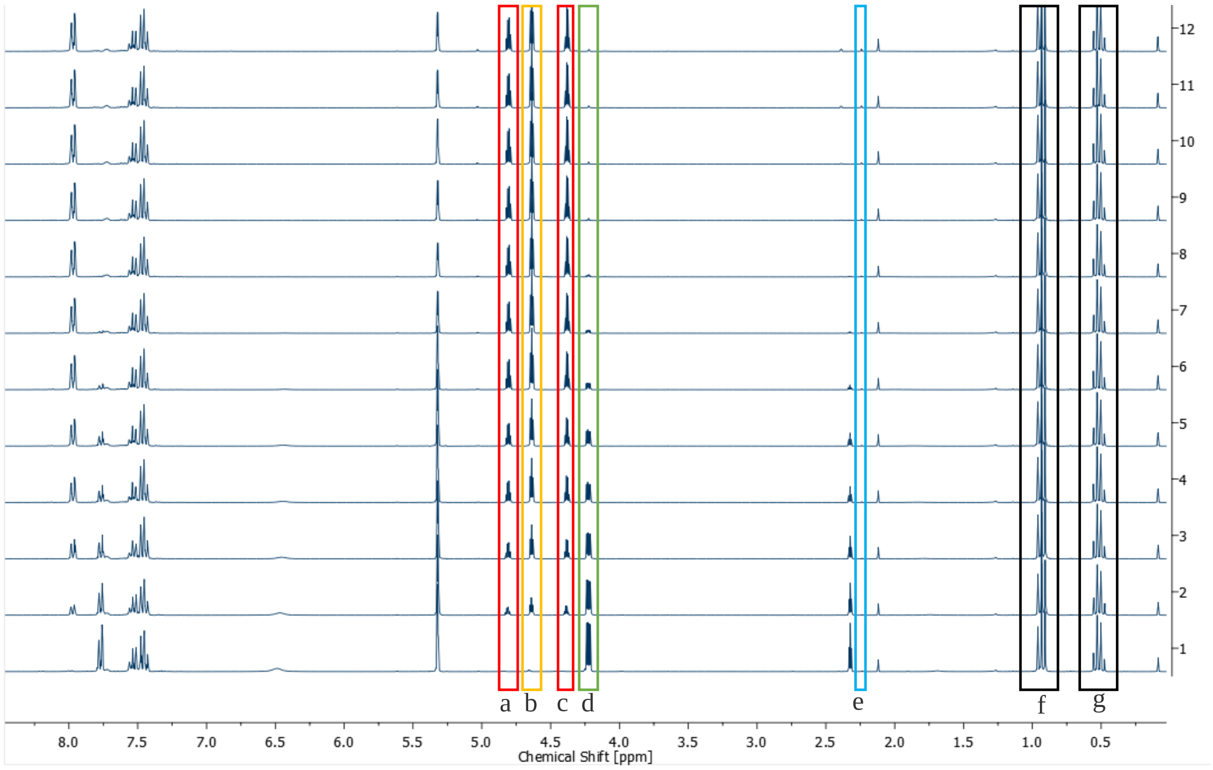

Figure S1: Periodic ^{1}H NMR measurements (300 MHz, CD_2Cl_2) of $\mathbf{7^{BArF}}$ (2 mol %) between 12 min after reaction start and ca. 18 h. Signals a, c and b (red, red, yellow) belong to the product, signal d (green) belongs to the starting material, signal e (blue) belongs to the CH_3 group of the aromatic product and signals f and g (black) belong to the standard (tetraethylsilane). Cf. **Scheme S1**. In cases when there was more than one signal per molecule, the average of all signals (indicated in **Scheme S1** and this figure) was taken.

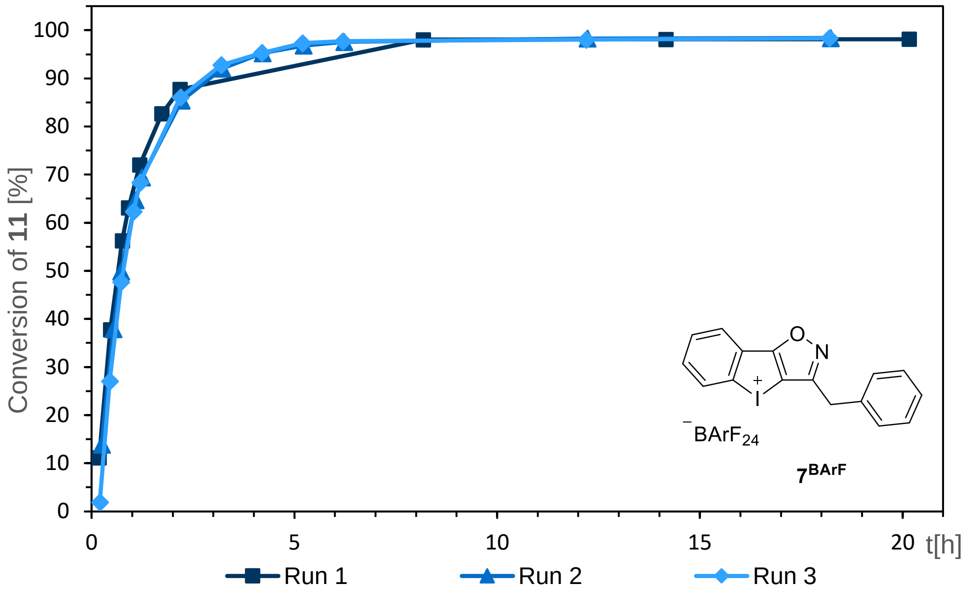

Figure S2: Conversion of propargylic amide **11** [%] by the activation of chloro(triphenylphosphine)gold by iodoloisoxazolium $\mathbf{7}^{\mathbf{BArF}}$. Catalyst system loading of 2 mol %.

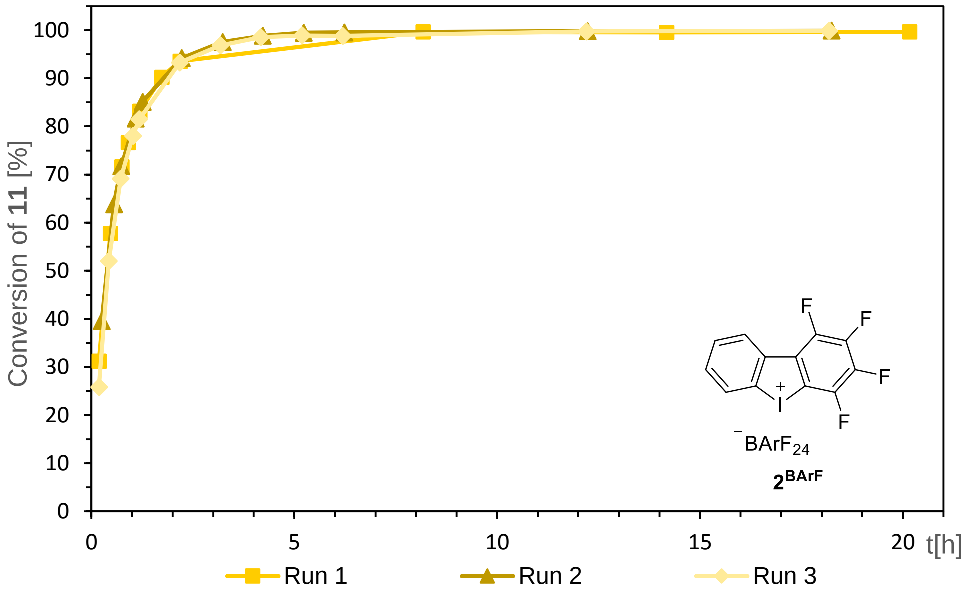

Figure S3: Conversion of propargylic amide **11** [%] by the activation of chloro(tri-phenylphosphine)gold by tetrafluoroiodolium $\mathbf{2}^{\mathbf{BArF}}$. Catalyst system loading of 2 mol %.

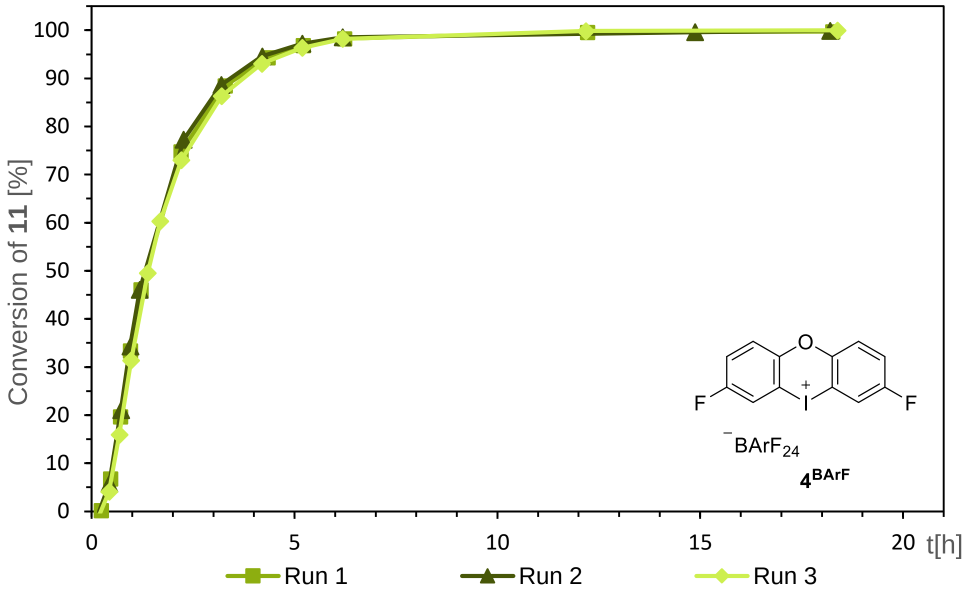

Figure S4: Conversion of propargylic amide **11** [%] by the activation of chloro(tri-phenylphosphine)gold by iodoxinium **4**$^{\mathbf{BArF}}$. Catalyst system loading of 2 mol %.

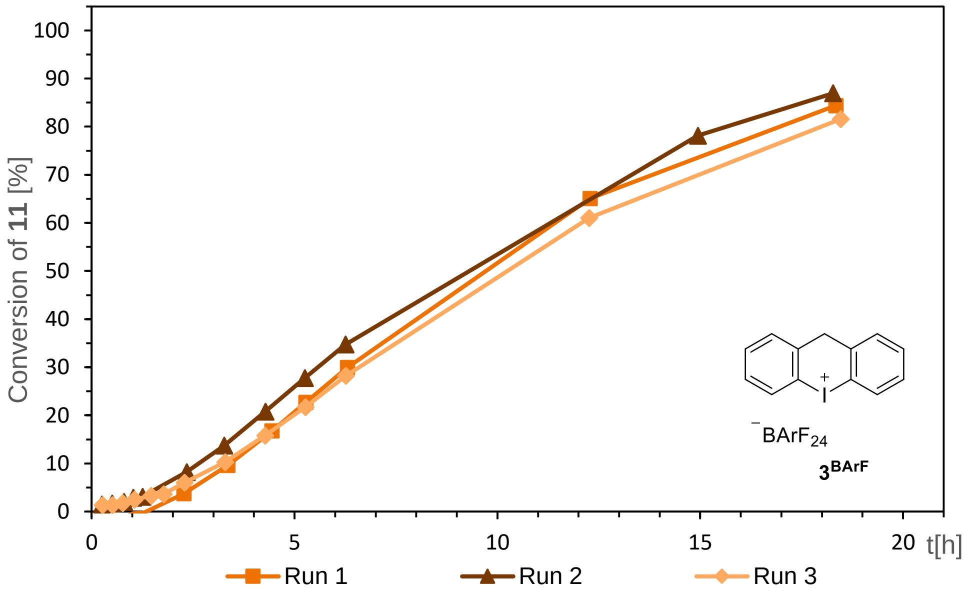

Figure S5: Conversion of propargylic amide **11** [%] by the activation of chloro(tri-phenylphosphine)gold by iodininium **3**$^{\mathbf{BArF}}$. Catalyst system loading of 2 mol %.

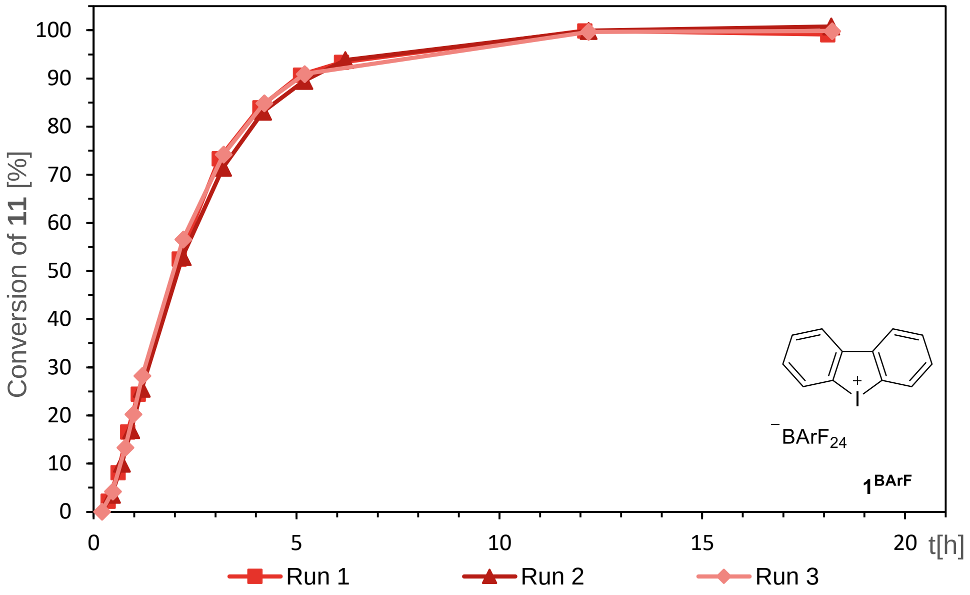

Figure S6: Conversion of propargylic amide **11** [%] by the activation of chloro(triphenylphosphine)gold by iodolium $\mathbf{1}^{\mathbf{BArF}}$. Catalyst system loading of 2 mol %.

3.4 Replica of performed catalytic experiments with 0.5 mol% catalyst system

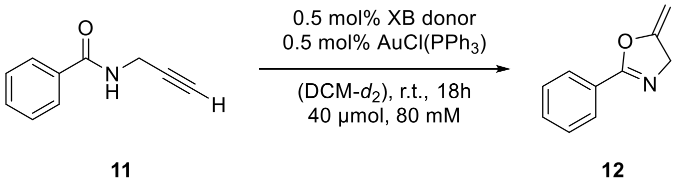

Scheme 2: Schematic representation of the activation of propargylic amide **11** with a catalyst system loading of 0.5 mol %.

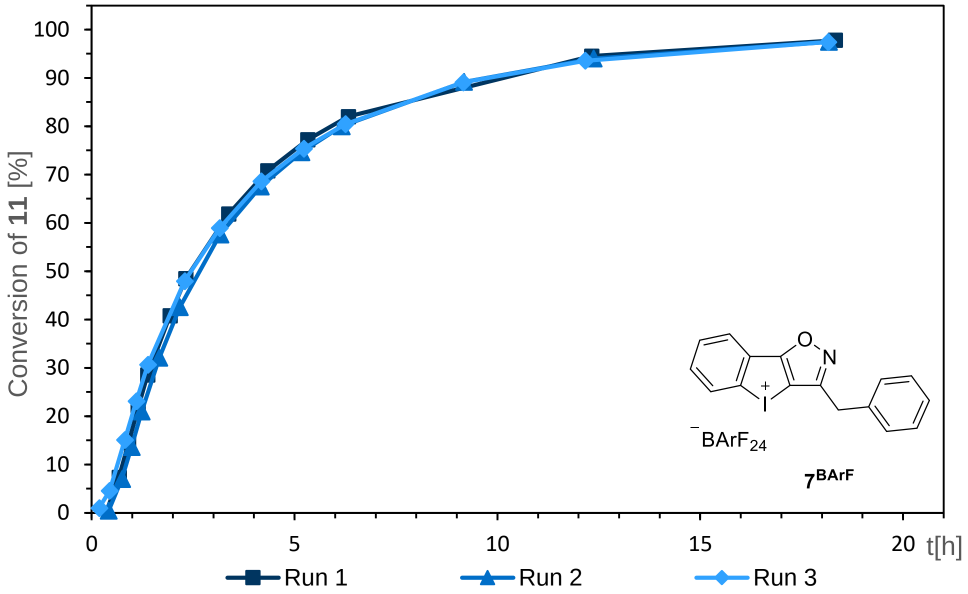

Figure S7: Conversion of propargylic amide **11** [%] by the activation of chloro(triphenylphosphine)gold by iodoloisoxazolium $\mathbf{7}^{\mathbf{BArF}}$. Catalyst system loading of 0.5 mol %.

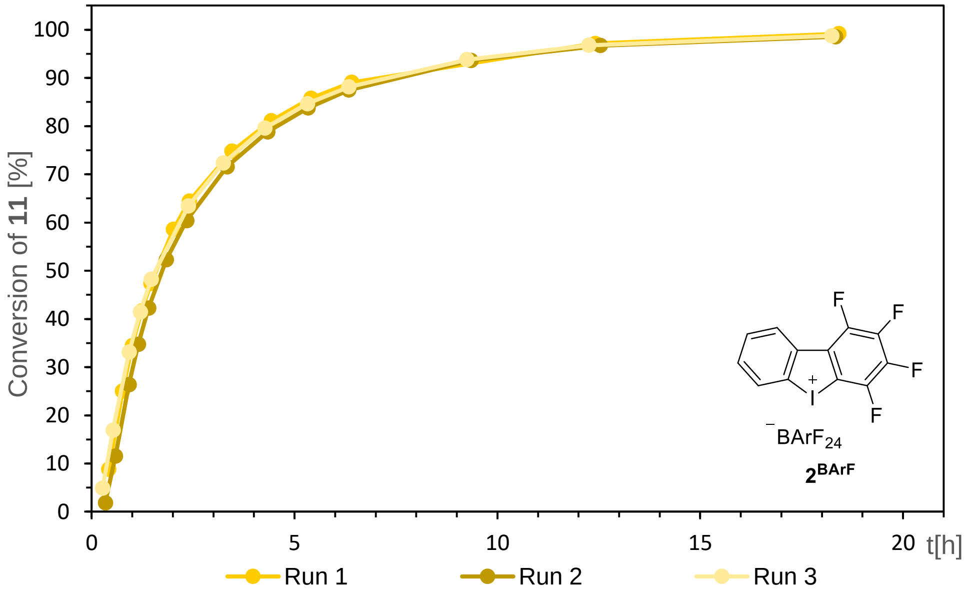

Figure S8: Conversion of propargylic amide **11** [%] by the activation of chloro(triphenylphosphine)gold by tetrafluoroiodolium $\mathbf{2}^{\mathbf{BArF}}$. Catalyst system loading of 0.5 mol %.

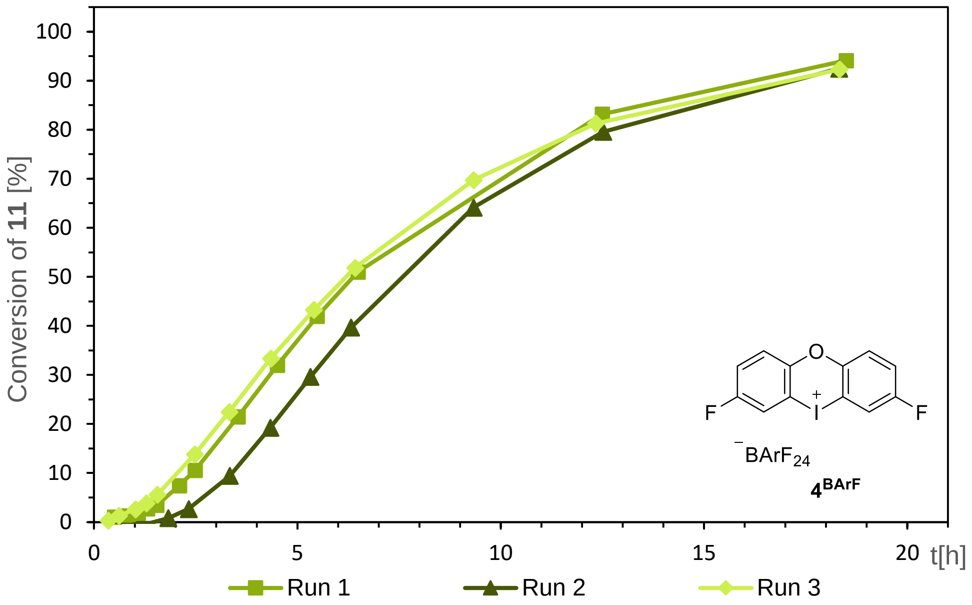

Figure S9: Conversion of propargylic amide **11** [%] by the activation of chloro(tri-phenylphosphine)gold by iodoxinium $\mathbf{4^{BArF}}$. Catalyst system loading of 0.5 mol %.

3.5 Determination of turnover frequencies (TOFs) of 7^{BArF} and 2^{BArF}

Pseudo-first-order reaction kinetics were assumed for the present reaction:

$$[S]_t = [S]_0 \cdot e^{-k \cdot t} \quad (1)$$

With:

$$[S]_t = concentration\ of\ the\ starting\ material\ at\ time\ t\ \left[\frac{mol}{l}\right]$$

$$[S]_0 = initial\ concentration\ of\ starting\ material\ \left[\frac{mol}{l}\right] = 0.08\ \left[\frac{mol}{l}\right]$$

$$k = rate\ constant\ [h^{-1}]$$

$$t = time\ [h]$$

Which forms to:

$$k = -\ln\left(\frac{[S]_t}{[S]_0}\right)/t \quad (2)$$

On the example of the 3rd run of 2 mol % iodoloisoxazolium $\mathbf{7}^{\mathbf{BArF}}$ (cf. Figure S2) the TOF [h^{-1}] is calculated:

To determine the rate constant k the negative *ln* of the concentration ratio $[S]_t/[S]_0$ is plotted against the time:

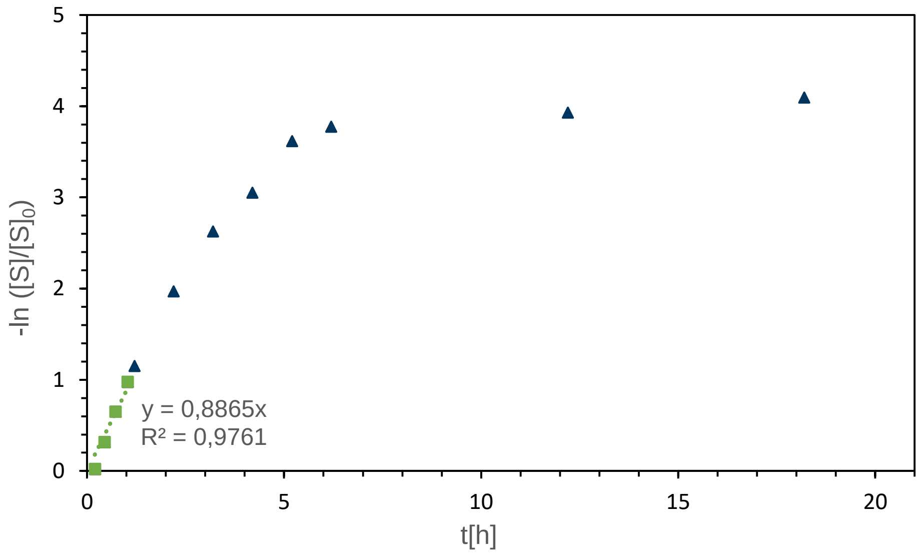

Figure S9: Logarithmic plot against of the activation reaction with 2 mol % iodoloisoxazolium $\mathbf{7}^{\mathbf{BArF}}$ (cf. Figure S2). Only the first four data points (green squares) were assumed as linear and were therefore taken for the determination of the TOF. The origin of the regression is set as 0/0. The slope of the regression equals *k*.

With $k = 0.89\ h^{-1}$ in this case (cf. Figure S9) the initial reaction rate r_0 can be determined:

$$r_0 = [S]_0 \cdot k \quad (3)$$

$$r_0(\mathbf{7}^{\boldsymbol{BArF}} - Run\ 3) = 0.71\ \frac{mol}{l} \cdot h^{-1}$$

From the initial reaction rate r_0 the TOF can be calculated:

$$TOF = \frac{r_0}{[Cat]} \quad (4)$$

$$TOF(\mathbf{7}^{\boldsymbol{BArF}} - Run\ 3) = 44\ h^{-1}$$

With:

$$[Cat]_{2\ mol\%} = Concentration\ of\ the\ Catalyst\ System\ \left[\frac{mol}{l}\right] = 0.0016\ \frac{mol}{l}$$

In this way following TOFs were determined:

Table S1: Determined TOFs [h^{-1}] per run, average value (av.) and calculated standard deviation (st.d.).

	Run (2 mol %)					Run (0.5 mol %)*					Total	
	1	2	3	av.	st.d.	1	2	3	av.	st.d.	av	st.d.
**7**$^{\mathbf{BArF}}$	53.93	46.9	44.3	**48.4**	**4.1**	-	-	-	-	-	**48.4**	**4.1**
**2**$^{\mathbf{BArF}}$	83.61	82.7	77.9	81.4	2.5	82.5	65.0	85.2	77.68	9.0	**80.0**	**7.0**
**4**$^{\mathbf{BArF}}$	17.22	18.11	15.41	**16.9**	**1.1**	-	-	-	-	-	**16.9**	**1.1**

*for **7**$^{\mathbf{BArF}}$ and **4**$^{\mathbf{BArF}}$ a sigmoidal curve was observed in the beginning of the reaction, therefore the TOF could not be determined.

3.6 Control experiments

The following control experiments were conducted:

Table S2: Control experiments performed in CD_2Cl_2 with respective concentrations.

Entry	XB donor	Chloro(triphenylphosphine) gold	Propargylic amide **11**	Reaction within 18 h?
1	-	1.6 mM	80 mM	no
2	**7** 1.6 mM	-	80 mM	no
3	**2** 1.6 mM	-	80 mM	no
4	**4** 1.6 mM	-	80 mM	no
5	**3** 1.6 mM	-	80 mM	no
6	**1** 1.6 mM	-	80 mM	no
7	**7** 5 mM	5 mM	-	Degradation of anion
8	**2** 5 mM	5 mM	-	“
9	**4** 5 mM	5 mM	-	“
10	**3** 5 mM	5 mM	-	“
11	**1** 5 mM	5 mM	-	“

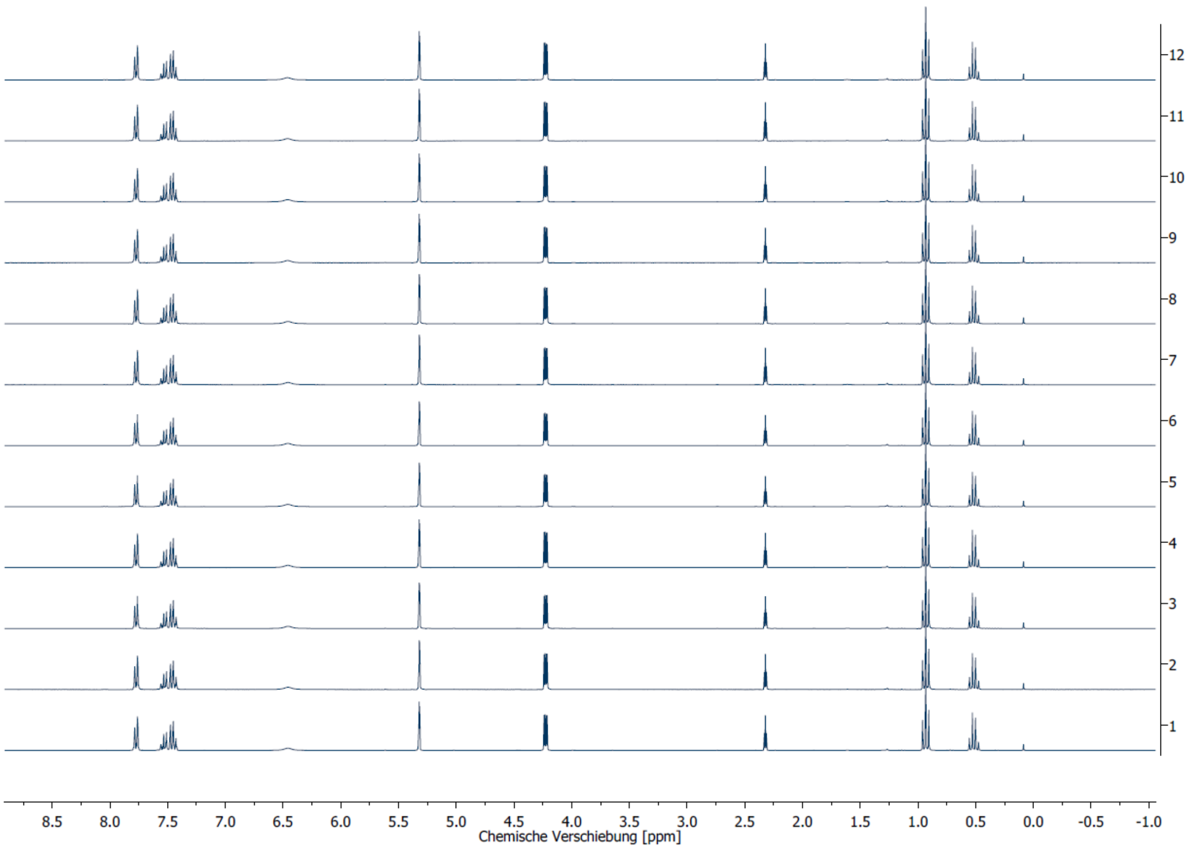

Figure S10: ^{1}H NMR spectra (300 MHz, CD_2Cl_2) of propargylic amide **11** (80 mM) and chloro(triphenylphosphine)gold (1.6 mM), measured periodically over 18 h (cf. Table S2, entry 1).

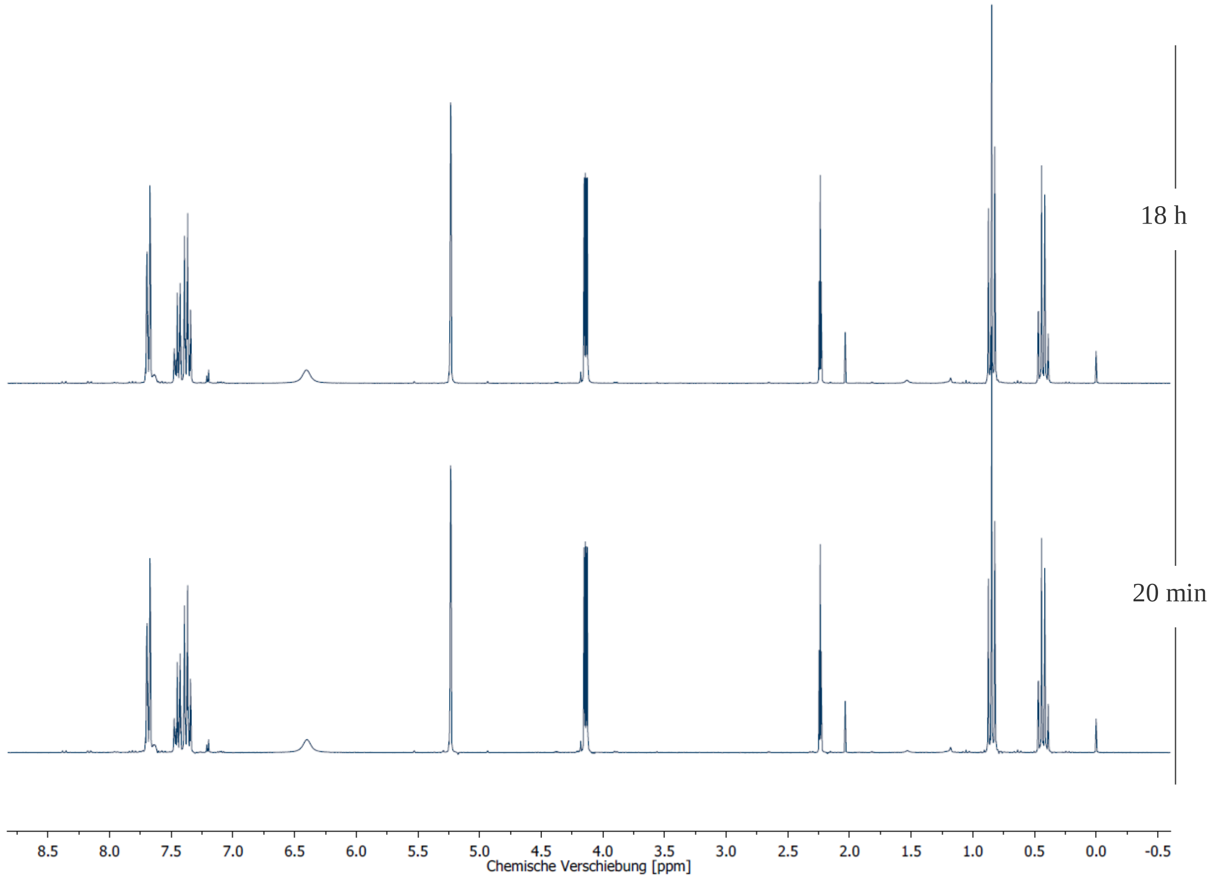

Figure S11: ^{1}H NMR spectra (300 MHz, CD_2Cl_2) of propargylic amide **11** (80 mM) and iodoloisoxazolium $\mathbf{7}^{\mathbf{BArF}}$ (1.6 mM) measured ca. 20 min after addition of the XB donor (bottom) and after 18 h (top; cf. Table S2, entry 2).

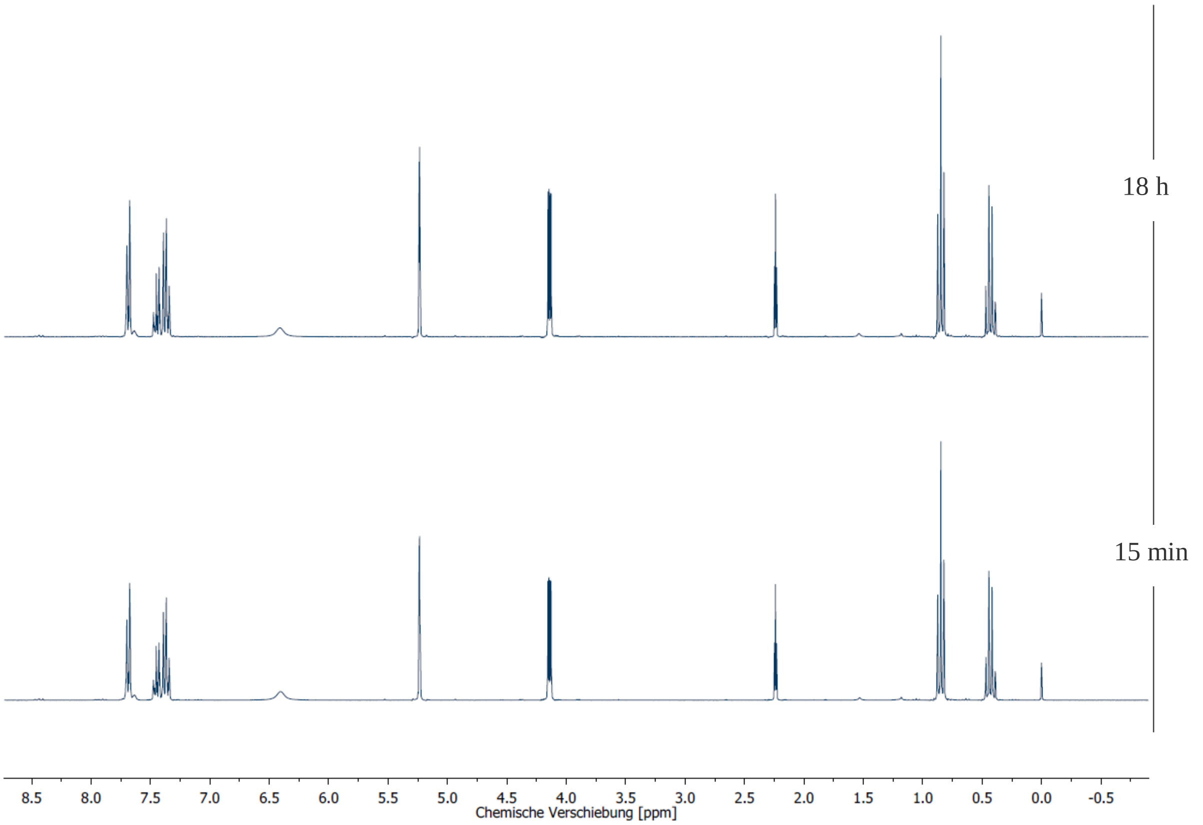

Figure S12: ^{1}H NMR spectra (300 MHz, CD_2Cl_2) of propargylic amide **11** (80 mM) and tetrafluoroiodolium $\mathbf{2}^{\mathbf{BArF}}$ (1.6 mM) measured ca. 15 min after addition of the XB donor (bottom) and after 18 h (top; cf. Table S2, entry 3).

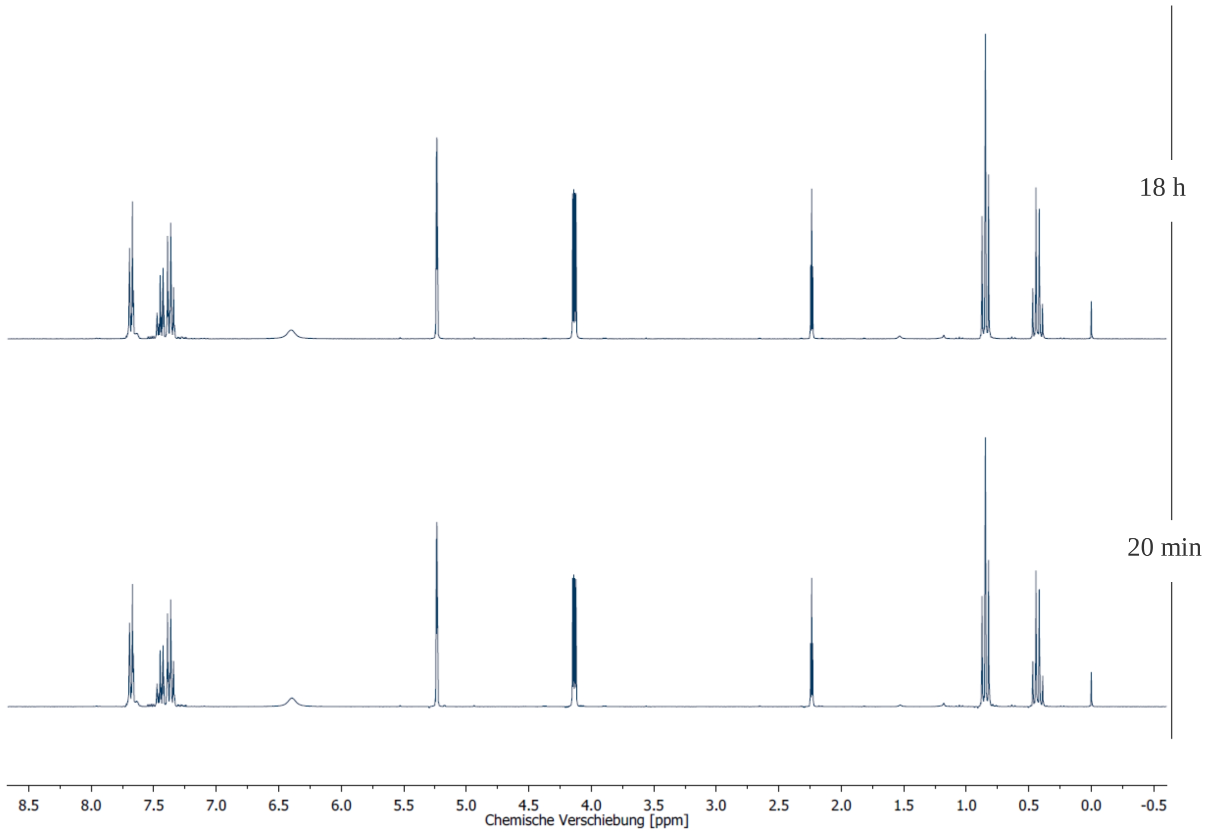

Figure S13: ^{1}H NMR spectra (300 MHz, CD_2Cl_2) of propargylic amide **11** (80 mM) and iodoxinium $\mathbf{4}^{\mathbf{BArF}}$ (1.6 mM) measured ca. 20 min after addition of the XB donor (bottom) and after 18 h (top; cf. Table S2, entry 4).

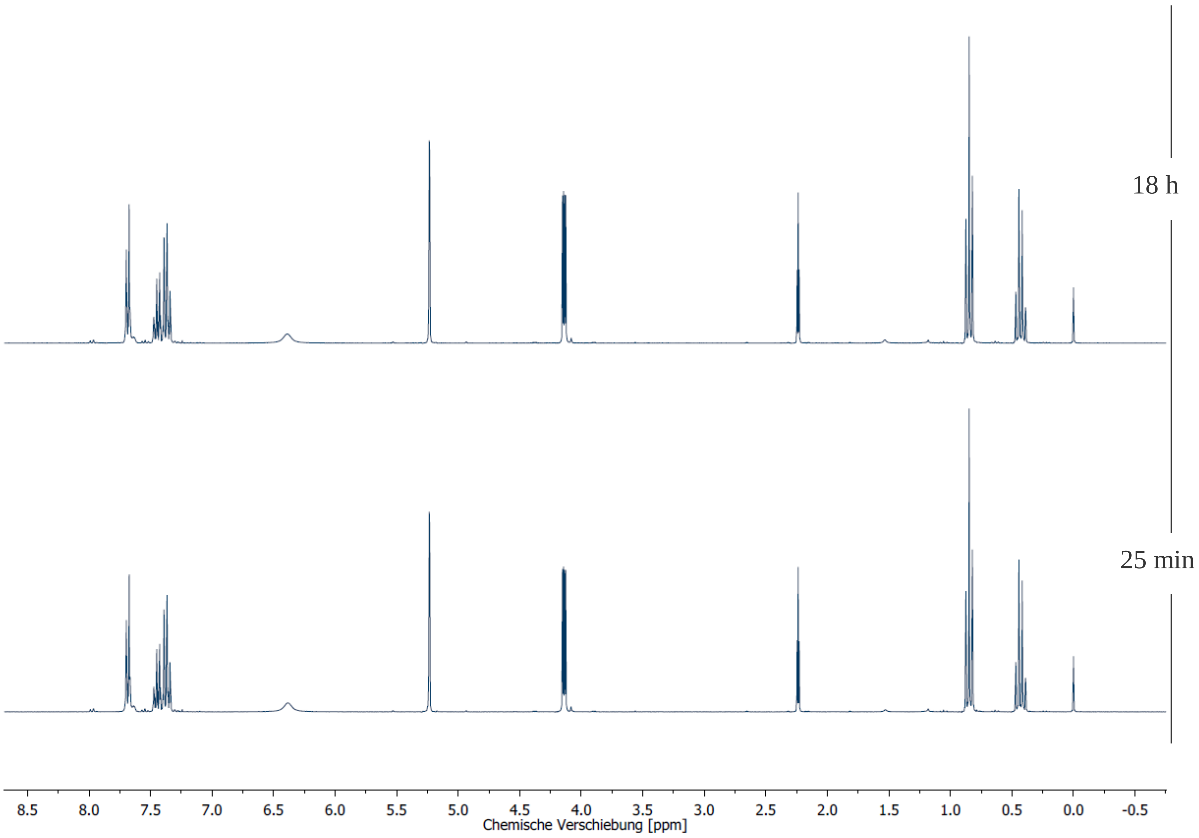

Figure S14: ^{1}H NMR spectra (300 MHz, CD_2Cl_2) of propargylic amide **11** (80 mM) and iodininium $\mathbf{3^{BArF}}$ (1.6 mM) measured ca. 25 min after addition of the XB donor (bottom) and after 18 h (top; cf. Table S2, entry 5).

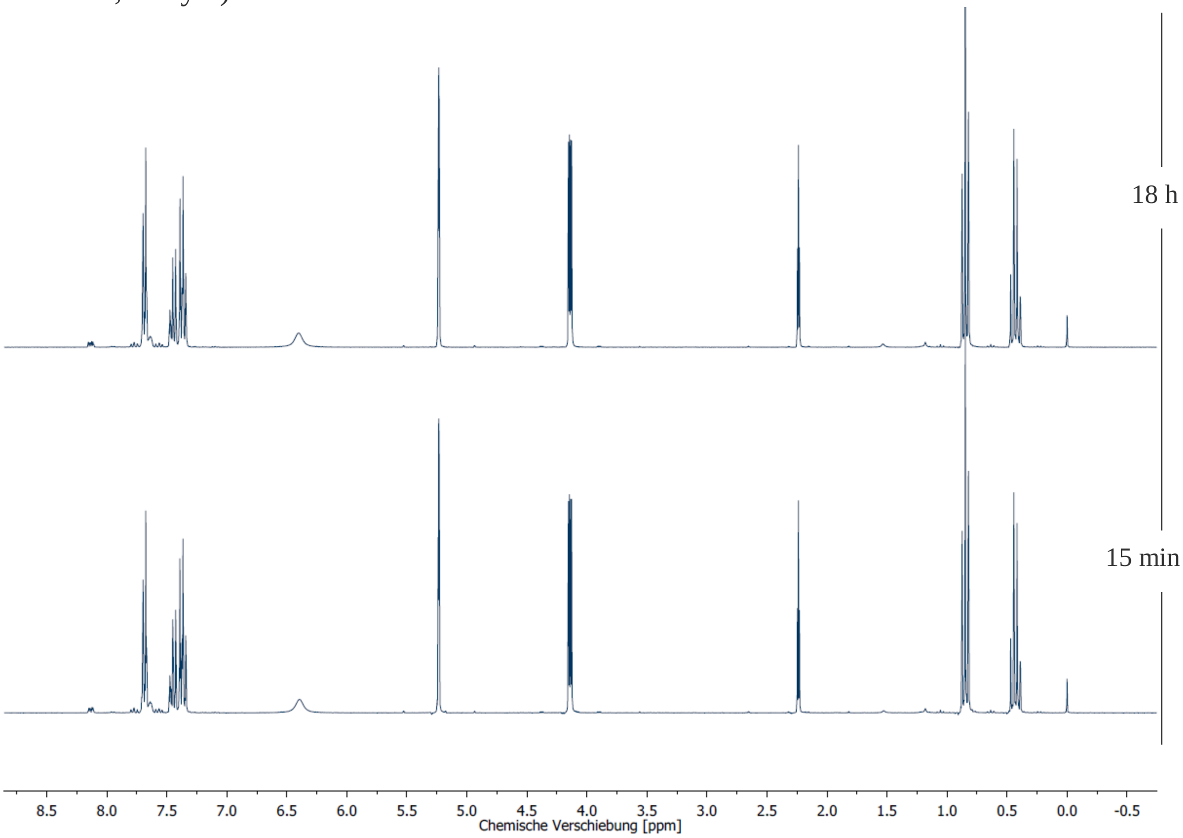

Figure S14: ^{1}H NMR spectra (300 MHz, CD_2Cl_2) of propargylic amide **11** (80 mM) and iodolium $\mathbf{1^{BArF}}$ (1.6 mM) measured ca. 15 min after addition of the XB donor (bottom) and after 18 h (top; cf. Table S2, entry 6).

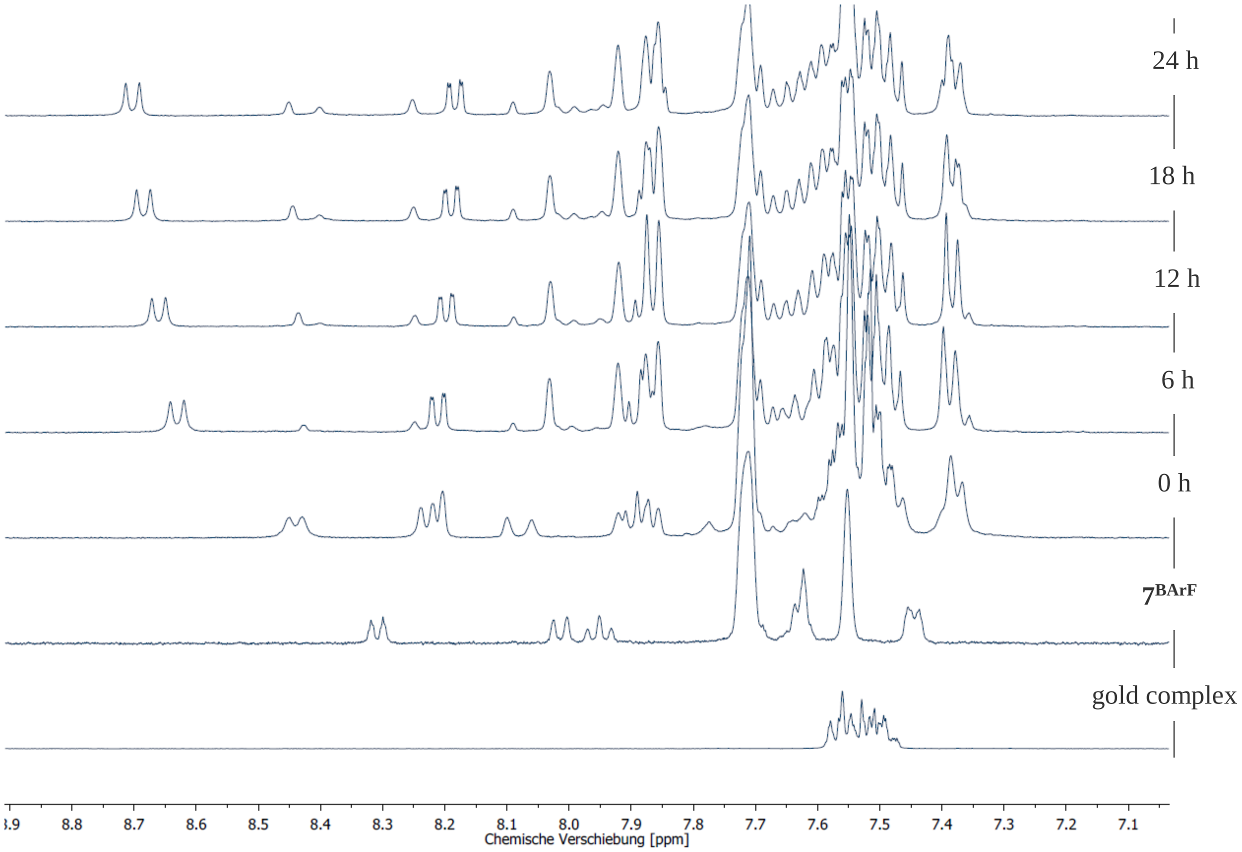

Figure S15: ^{1}H NMR spectra (400 MHz, CD_2Cl_2) of chloro(triphenylphosphine)gold (5 mM) and iodoloisoxazolium $\mathbf{7}^{\mathbf{BArF}}$ (5 mM) measured periodically (top; cf. Table S2, entry 7).

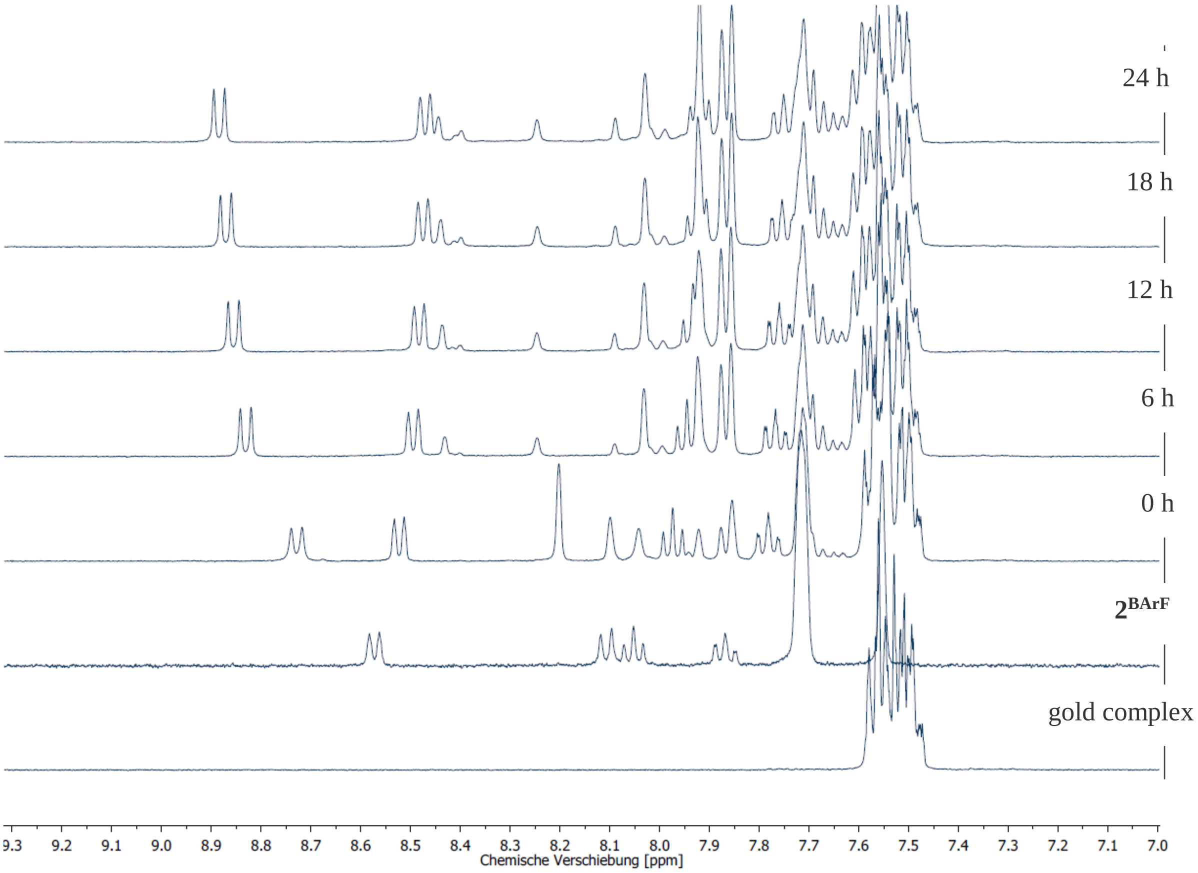

Figure S16: ^{1}H NMR spectra (400 MHz, CD_2Cl_2) of chloro(triphenylphosphine)gold (5 mM) and tetrafluoroiodolium $\mathbf{2}^{\mathbf{BArF}}$ (5 mM) measured periodically (top; cf. Table S2, entry 8).

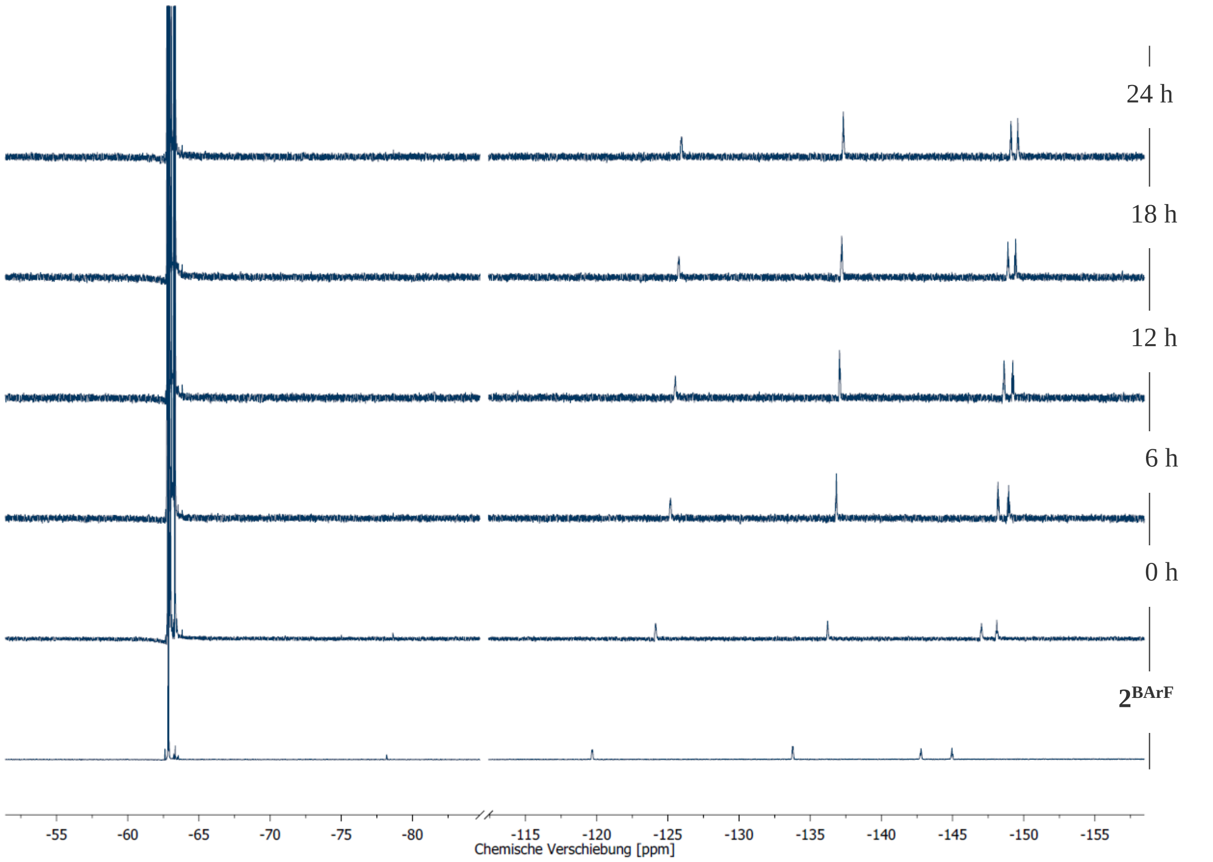

Figure 17: ^{19}F NMR spectra (400 MHz, CD_2Cl_2) of chloro(triphenylphosphine)gold (5 mM) and tetrafluoroiodolium **2**$^{\mathbf{BArF}}$ (5 mM) measured periodically (top; cf. Table S2, entry 8).

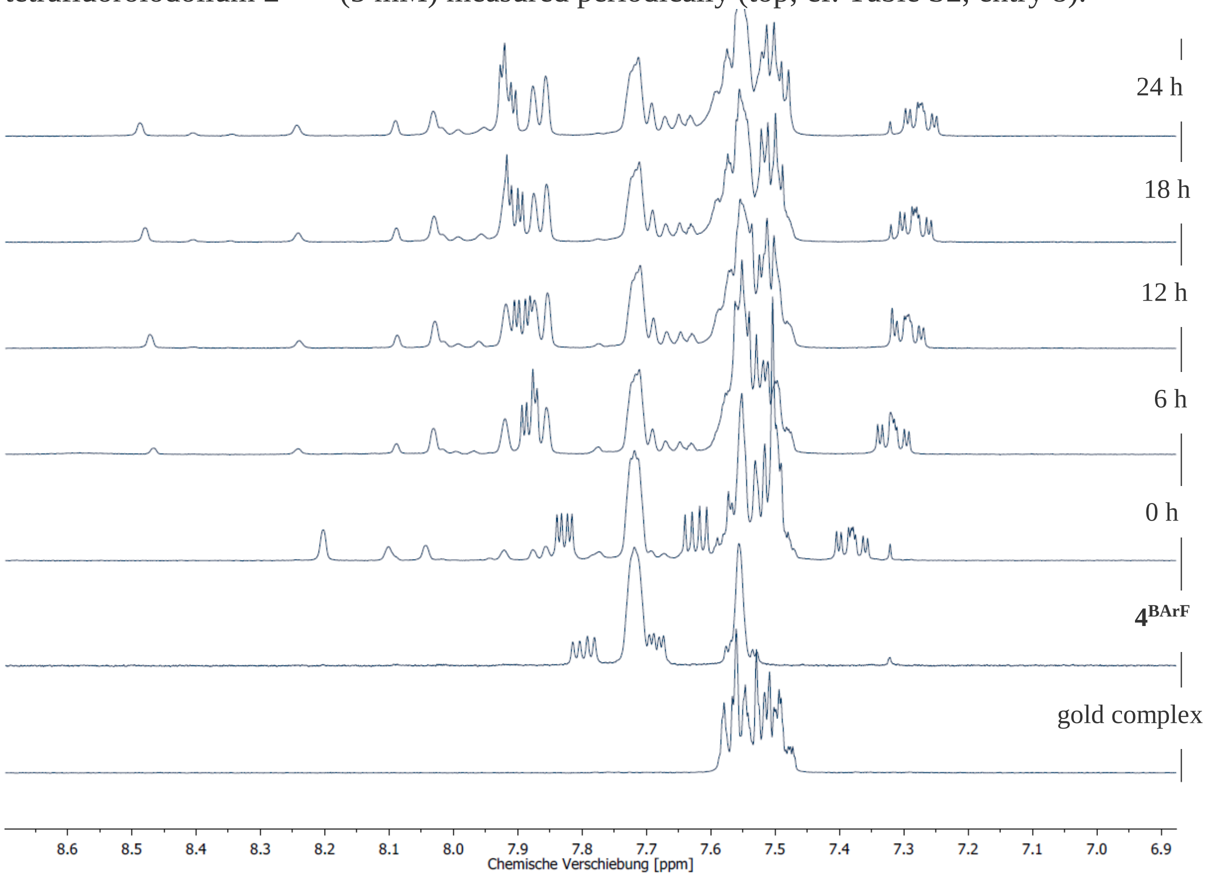

Figure S18: ^{1}H NMR spectra (400 MHz, CD_2Cl_2) of chloro(triphenylphosphine)gold (5 mM) and iodoxinium **4**$^{\mathbf{BArF}}$ (5 mM) measured periodically (top; cf. Table S2, entry 9).

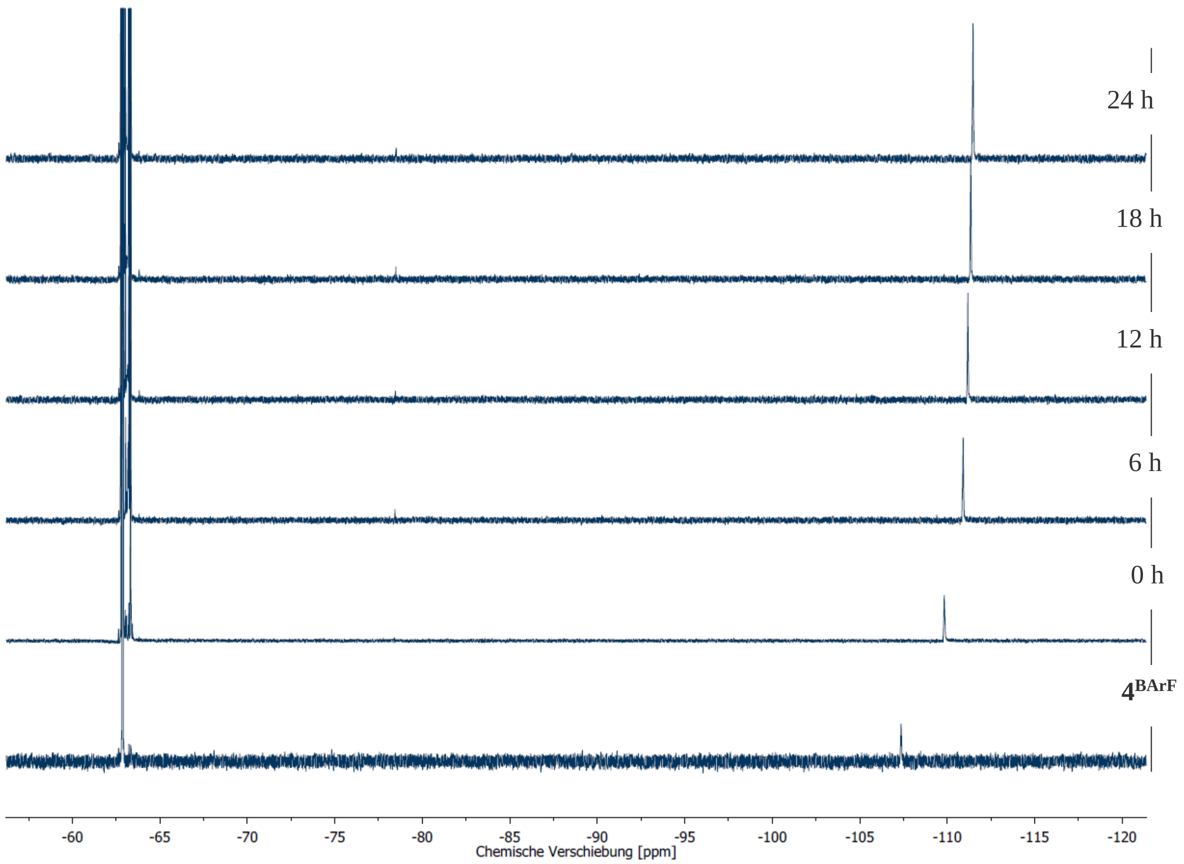

Figure S19: ^{19}F NMR spectra (400 MHz, CD_2Cl_2) of chloro(triphenylphosphine)gold (5 mM) and iodoxinium **4**$^{\mathbf{BArF}}$ (5 mM) measured periodically (top; cf. Table S2, entry 9).

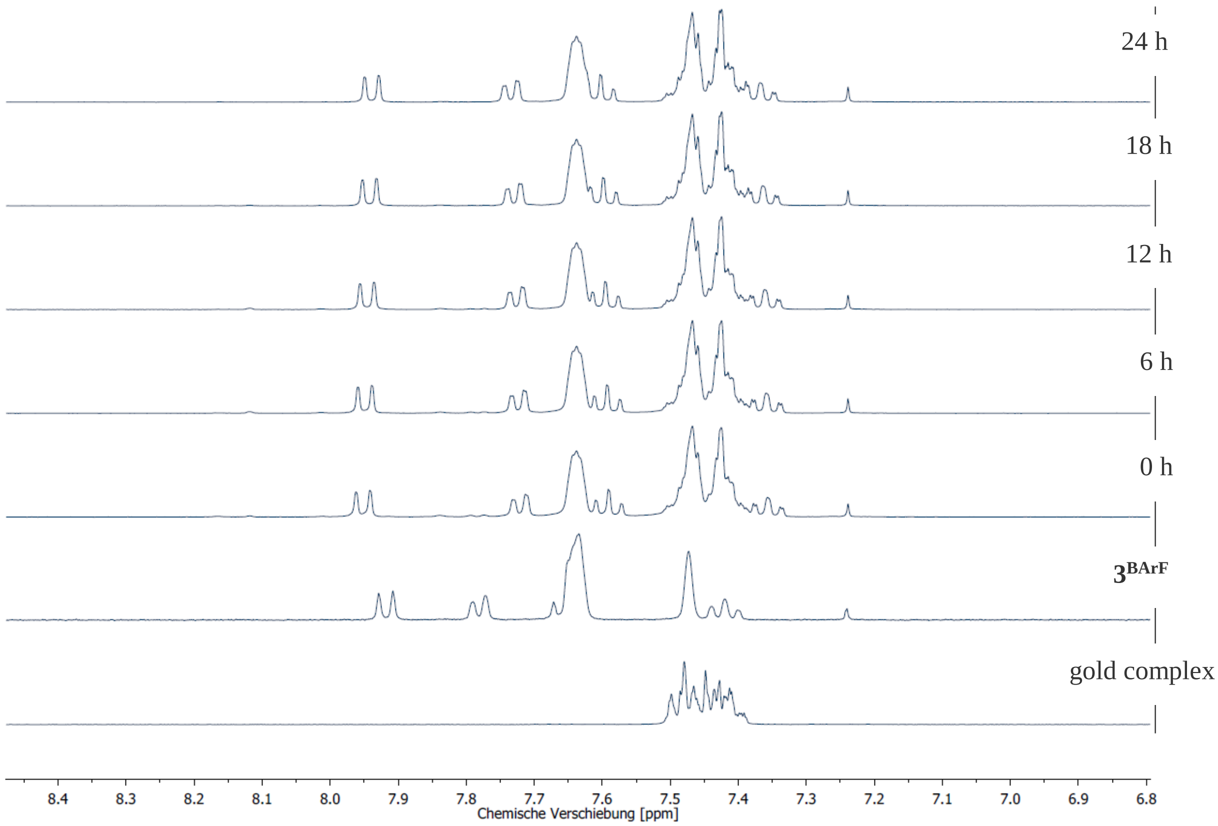

Figure S20: ^{1}H NMR spectra (400 MHz, CD_2Cl_2) of chloro(triphenylphosphine)gold (5 mM) and iodininium **3**$^{\mathbf{BArF}}$ (5 mM) measured periodically (top; cf. Table S2, entry 10).

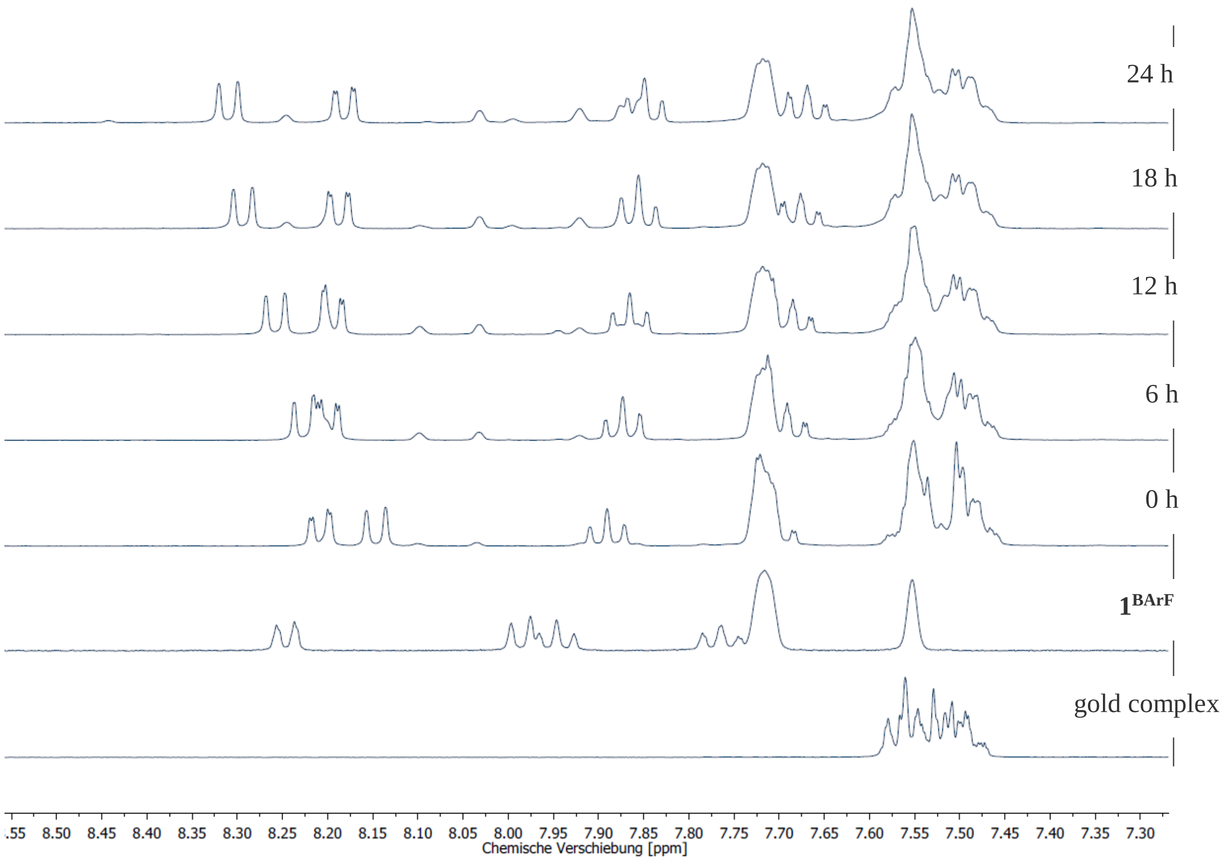

Figure S21: ^{1}H NMR spectra (400 MHz, CD_2Cl_2) of chloro(triphenylphosphine)gold (5 mM) and iodolium $\mathbf{1}^{\mathbf{BArF}}$ (5 mM) measured periodically (top; cf. Table S2, entry 11).

4. Crystallography

4.1 Crystallization of 7^{Br}

X-ray grade crystals of the bromide salt $\mathbf{7}^{\mathbf{Br}}$ were grown according to the following procedure: 200 µL each of a 25 mM stock solution of $\mathbf{7}^{\mathbf{OTf}}$, a 25 mM stock solution of α-methylbenzyl bromide (CAS: 585-71-7) and a 50 mM stock solution of H_2O in dry CD_3CN were mixed in an NMR tube. After some days crystals had formed which were used for crystal structure determination.

4.2 Crystal structure data

Crystal structure determination was carried out on a Rigaku Synergy dual source device, with Cu micro focus sealed tubes (Cu Kα) using mirror monochromators and a HyPix-6000HE: Hybrid photon counting X-ray detector. Crystals were mounted in Hampton CryoLoops using Parabar/Paratone or GE/Bayer silicone grease. Data was recorded and reduced using the CrysalisPro Software [6]. Structures were solved using WinGX [7] in combination with ShelXT and refined with shelXle and ShelXL [8,9]. Tables for the publication were generated using a modified version of Ciftab. Analysis and visualization of the structures was done with Diamond 4 [10].

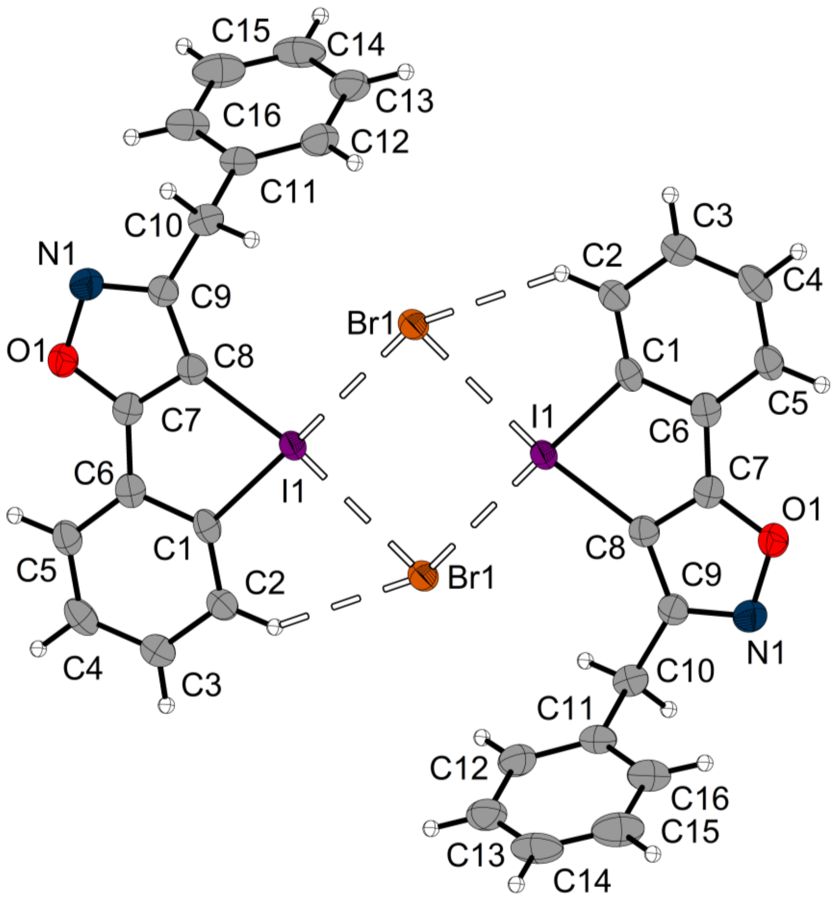

Figure S22: Crystal structure of **7^{Br}** showing the halogen bonding dimer.

Table S3: Crystal data and structure refinement for **7^{Br}**.

Compound	**7^{Br}**
CCDC-Number	2362114
Empirical formula	C16 H11 Br I N O
Formula weight [g/mol]	440.07
Crystal system	Monoclinic
Space group	$P2_1/n$ (14)
Lattice parameters [Å]	
a	5.47070(10)
b	10.91390(10)
c	24.3668(3)
α	90
β	95.6010(10)
γ	90
Density [g/cm^3]	2.019
Crystal size [mm^3]	0.159 x 0.137 x 0.063
Volume [Å^3]	1447.91(3)
Z	4
Temperature [K]	169.99(10)
Diffraction Device	XtaLAB Synergy, Dualflex, HyPix
Radiation Type	1.54184 Å (Cu K/ micro-focus sealed X-ray tube)
F(000)	840
Absorption coefficient [mm^{-1}]	20.56
Absorption correction	Gaussian
Measurement range	3.6 - 66.5
Index range	-6 < h < 6
	-12 < k < 12
	-27 < l < 28
Measured reflexes	13403
Independent	2560
Observed	2434
R(int)	0.055
Completeness (%) / theta (°)	100.0 / 66.482
Transmission (min / max)	0.186 / 0.589
R1 (observed/all)	0.0267 / 0.0286
wR2 (observed/all)	0.0725 / 0.0738
GooF = S	1.048
Measured reflexes	-0.808 / 0.754
Independent	2362114
Observed	oc1h-dlr-cat-012-c
R(int)	C16 H11 Br I N O
Completeness (%) / theta (°)	440.07
Transmission (min / max)	Monoclinic
R1 (observed/all)	$P2_1/n$ (14)
wR2 (observed/all)	
GooF = S	5.47070(10)
Rest electron density max./min. [e-/Å^3]	10.91390(10)

Table S4: Atomic coordinates (× 10^4) and equivalent isotropic displacement parameters (Å^2 × 10^3) for **7^{Br}**.

	x	y	z	U(eq)
I(1)	0.73431(3)	0.15090(2)	0.50019(2)	0.02363(11)
O(1)	0.1352(5)	0.3653(2)	0.45188(11)	0.0320(6)
N(1)	0.0936(5)	0.2955(3)	0.40203(12)	0.0341(7)
C(1)	0.6463(6)	0.2935(3)	0.55510(14)	0.0266(7)
C(2)	0.7697(7)	0.3165(3)	0.60563(14)	0.0309(7)
C(3)	0.6784(7)	0.4093(3)	0.63785(16)	0.0354(8)
C(4)	0.4695(7)	0.4750(3)	0.61845(16)	0.0362(8)
C(5)	0.3485(7)	0.4509(3)	0.56747(15)	0.0308(7)
C(6)	0.4356(7)	0.3590(3)	0.53448(16)	0.0279(8)
C(7)	0.3339(6)	0.3190(3)	0.48136(14)	0.0267(7)
C(8)	0.4273(6)	0.2246(3)	0.45363(13)	0.0266(7)
C(9)	0.2693(6)	0.2133(3)	0.40390(13)	0.0269(7)
C(10)	0.2765(7)	0.1256(3)	0.35648(15)	0.0322(8)
C(11)	0.4109(7)	0.1758(3)	0.30970(14)	0.0317(8)
C(12)	0.6049(7)	0.1111(4)	0.29100(16)	0.0417(9)
C(13)	0.7285(9)	0.1557(5)	0.24791(17)	0.0484(11)
C(14)	0.6578(9)	0.2649(5)	0.22324(16)	0.0501(11)
C(15)	0.4624(10)	0.3287(4)	0.24024(18)	0.0509(12)
C(16)	0.3379(8)	0.2854(4)	0.28321(16)	0.0400(9)
Br(1)	1.16928(6)	0.07117(3)	0.58085(2)	0.02889(12)
H(2)	0.912225	0.271212	0.618447	0.037
H(3)	0.759618	0.427343	0.673175	0.042
H(4)	0.409817	0.537559	0.64076	0.043
H(5)	0.206152	0.496425	0.55473	0.037
H(10A)	0.10601	0.105035	0.342039	0.039
H(10B)	0.357734	0.048954	0.370167	0.039
H(12)	0.653971	0.035359	0.307856	0.05
H(13)	0.861505	0.110802	0.235583	0.058
H(14)	0.744204	0.296405	0.94341	0.06
H(15)	0.41213	0.403258	0.222412	0.061
H(16)	0.202882	0.330175	0.29467	0.048

Table S5: Anisotropic displacement parameters (Å^2 × 10^3) for **7^{Br}**.

	U^{11}	U^{22}	U^{33}	U^{23}	U^{13}	U^{12}
I(1)	0.02519(15)	0.02235(16)	0.02405(16)	-0.00014(7)	0.00595(10)	0.00260(7)
O(1)	0.0323(14)	0.0304(13)	0.0333(14)	0.0020(10)	0.0038(11)	0.0061(10)
N(1)	0.0340(16)	0.0391(17)	0.0293(15)	0.0040(13)	0.0027(12)	0.0027(14)
C(1)	0.0294(17)	0.0195(16)	0.0324(17)	-0.0011(13)	0.0097(14)	0.0026(14)
C(2)	0.0325(18)	0.0335(18)	0.0275(17)	-0.0012(14)	0.0068(15)	0.0032(15)
C(3)	0.039(2)	0.037(2)	0.0305(18)	-0.0050(15)	0.0048(16)	0.0016(16)
C(4)	0.045(2)	0.0273(18)	0.038(2)	-0.0063(15)	0.0156(16)	0.0022(16)
C(5)	0.0331(18)	0.0269(18)	0.0336(18)	0.0018(14)	0.0095(15)	0.0058(14)
C(6)	0.0289(19)	0.0235(17)	0.0324(19)	0.0051(13)	0.0084(16)	0.0001(14)
C(7)	0.0261(16)	0.0242(16)	0.0302(17)	0.0059(13)	0.0049(14)	-0.0009(14)
C(8)	0.0275(16)	0.0266(16)	0.0266(16)	0.0033(13)	0.0064(13)	-0.0003(14)
C(9)	0.0258(16)	0.0300(18)	0.0260(16)	0.0066(14)	0.0080(13)	-0.0007(14)
C(10)	0.0331(19)	0.0337(18)	0.0293(18)	0.0022(14)	0.0004(15)	-0.0025(15)
C(11)	0.0350(19)	0.0358(18)	0.0237(17)	-0.0016(14)	0.0007(15)	-0.0055(16)
C(12)	0.041(2)	0.054(2)	0.0294(19)	0.0074(18)	0.0009(16)	0.004(2)
C(13)	0.037(2)	0.082(3)	0.027(2)	0.0057(18)	0.0050(17)	0.0010(19)
C(14)	0.057(3)	0.069(3)	0.0248(18)	0.0068(19)	0.0068(18)	-0.024(2)
C(15)	0.077(4)	0.039(2)	0.034(2)	0.0066(18)	-0.003(2)	-0.013(2)
C(16)	0.057(2)	0.033(2)	0.0289(17)	0.0000(15)	0.0004(17)	-0.0045(18)
Br(1)	0.0323(2)	0.0286(2)	0.02626(19)	-0.00029(13)	0.00495(14)	0.00382(14)

5. Literature

(1) Heinen, F.; Engelage, E.; Dreger, A.; Weiss, R.; Huber, S. M. *Angew. Chem. Int. Ed.* **2018**, *57*, 3830-3833; *Angew. Chem.* **2018**, *130*, 3892-3896. doi:10.1002/anie.201713012

(2) Caspers, L. D.; Spils, J.; Damrath, M.; Lork, E.; Nachtsheim, B. J. *J. Org. Chem.* **2020**, *85*, 9161–9178. doi:10.1021/acs.joc.0c01125

(3) Reinhard, D. L.; Heinen, F.; Stoesser, J.; Engelage, E.; Huber, S. M. *Helv. Chim. Acta* **2021**, *104*, e2000221. doi:10.1002/hlca.202000221

(4) Yuan, H.; Wang, M.; Xu, Z.; Gao, H. *Adv. Synth. Catal.* **2019**, *361*, 4386–4392. doi:10.1002/adsc.201900435

(5) Wipf, P.; Aoyama, Y.; Benedum, T. E. *Org. Lett.* **2004**, *6*, 3593–3595. doi:10.1021/ol0485058

(6) **Crysalis**, W. P. M. Meyer, A. Kowalski, A. Muszynski, A. Wieniewski, M. Pol, M. Przewozniczek, P. Stec, D. Bujnik, H. Kulza *et al.*, Rigaku Oxford Diffraction (1995-2018),. Crysalis, 2018

(7) Farrugia, L. J. *J. Appl. Crystallogr.* **1999**, *32*, 837–838. doi:10.1107/S0021889899006020

(8) Sheldrick, G. M. *Acta Cryst. A* **2008**, *64*, 112–122. doi:10.1107/S0108767307043930

(9) Hübschle, C. B.; Sheldrick, G. M.; Dittrich, B. *J. Appl. Crystallogr.* **2011**, *44*, 1281–1284. doi:10.1107/S0021889811043202

(10) **Diamond - Crystal and Molecular Structure Visualization**, Crystal Impact - Dr. H. Putz & Dr. K. Brandenburg GbR, Kreuzherrenstr. 102, 53227 Bonn, Germany. Diamond 4, **https://www.crystalimpact.de/diamond.**

6. Appendix – NMR spectra

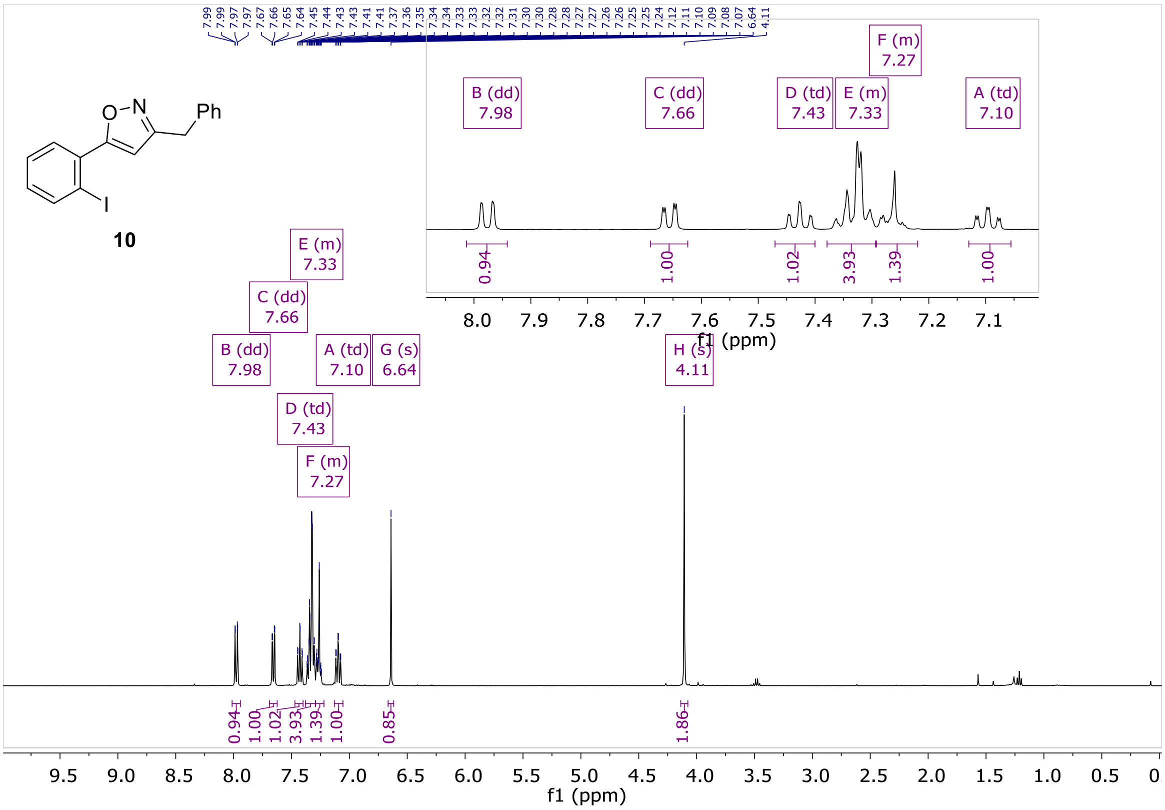

Figure S23: ^{1}H NMR spectrum of precursor **10** in $CDCl_3$.

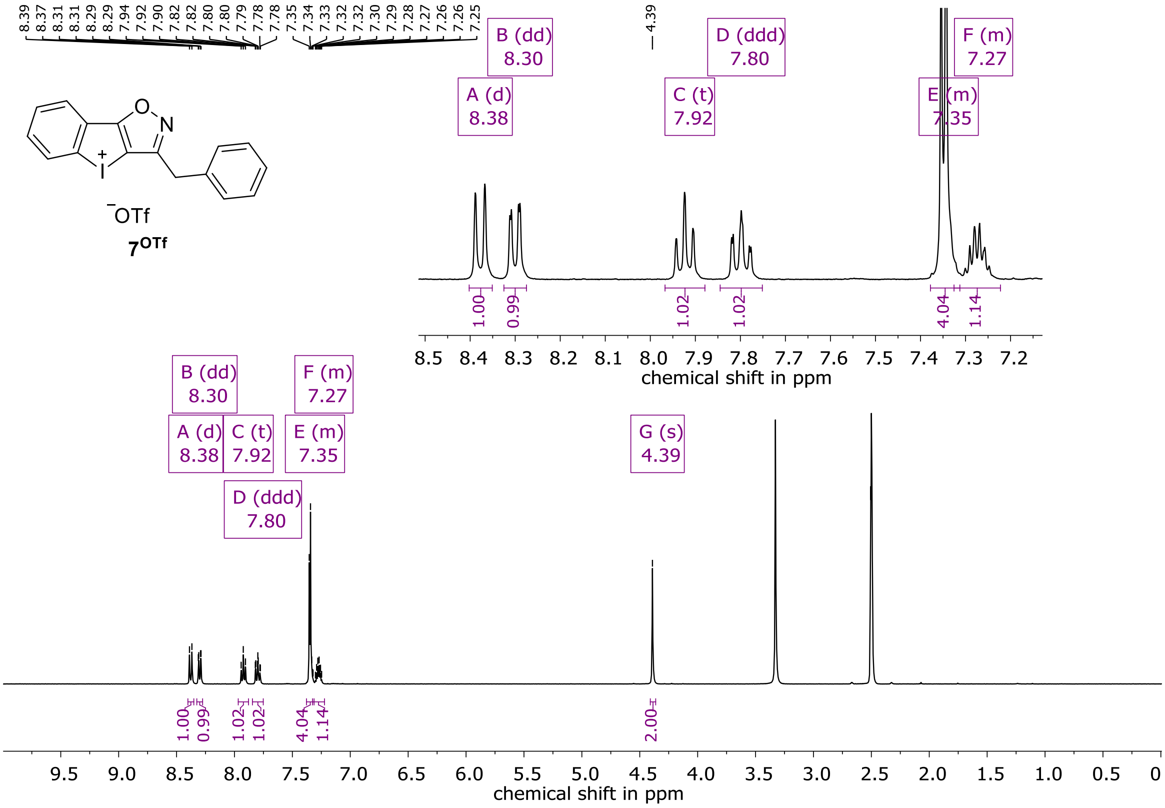

Figure S24: ^{1}H NMR spectrum of iodoloisoxazolium $\mathbf{7^{OTf}}$ in DMSO-d_6.

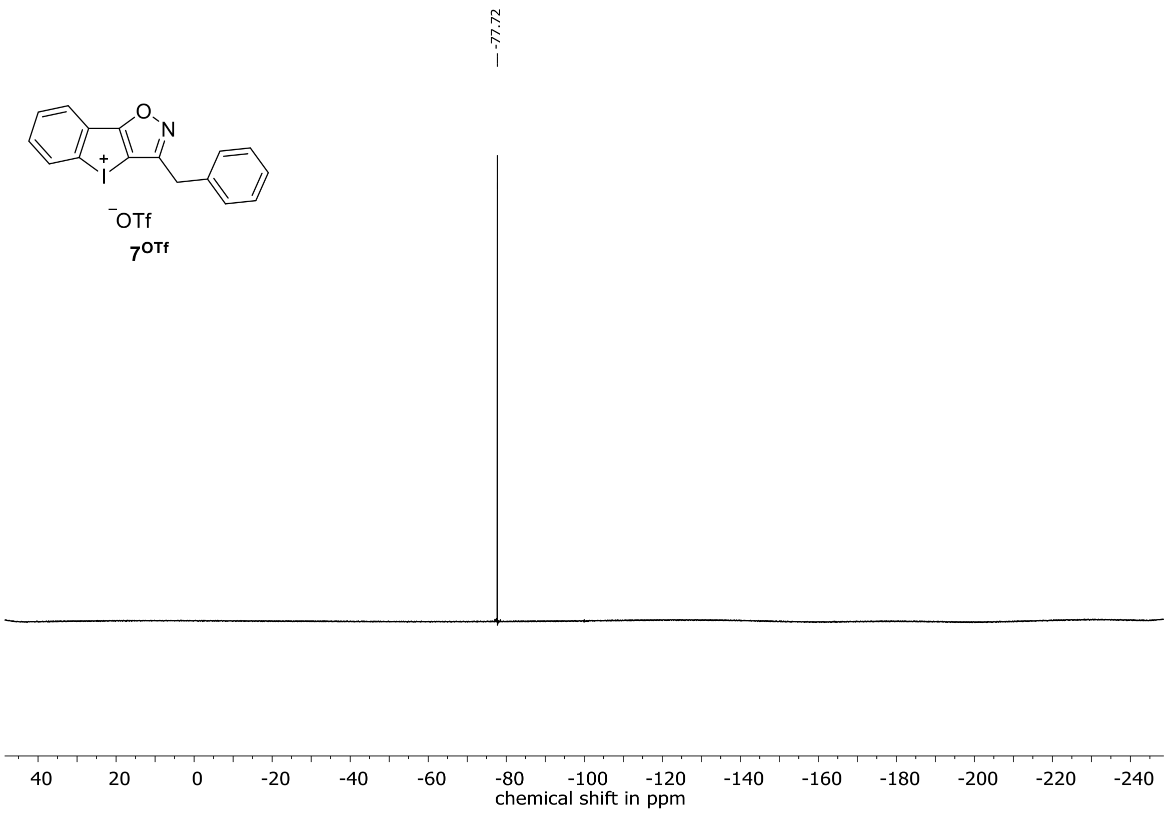

Figure S25: ^{19}F NMR spectrum of iodoloisoxazolium $\mathbf{7^{OTf}}$ in DMSO-d_6.

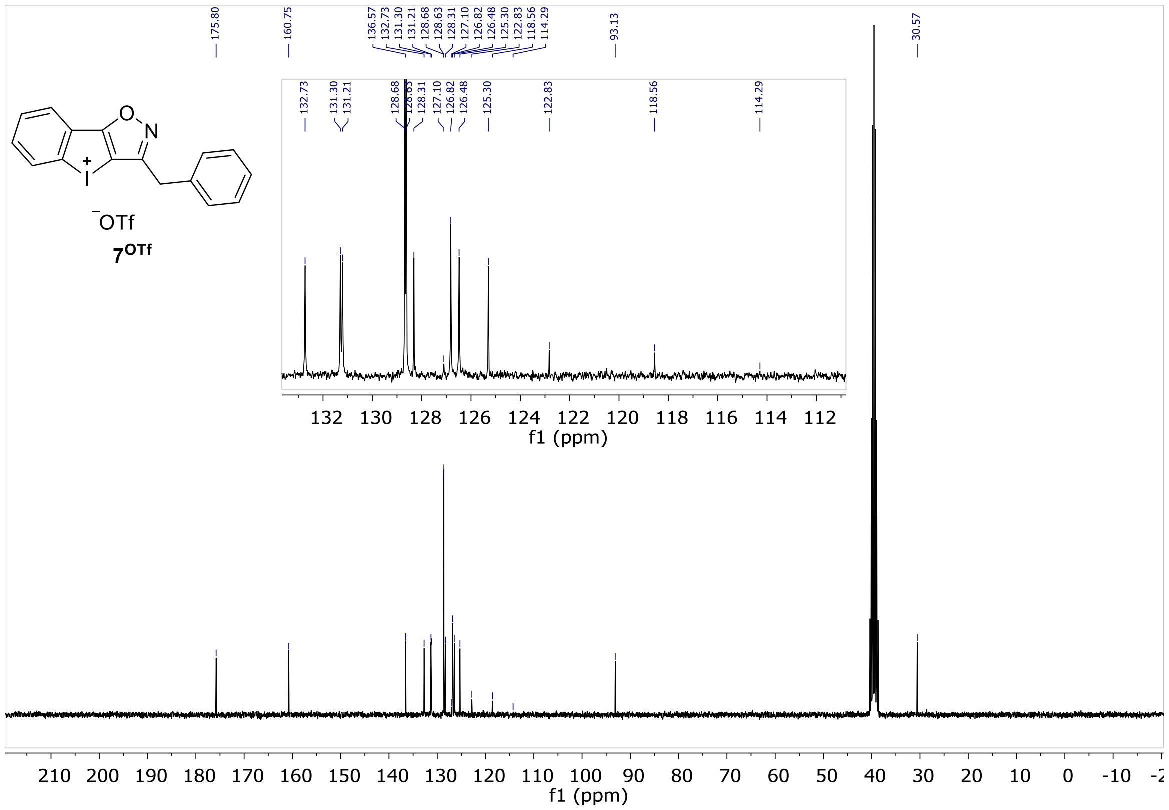

Figure S26: ^{13}C{^{1}H} NMR spectrum of iodoloisoxazolium $\mathbf{7^{OTf}}$ in DMSO-d_6.

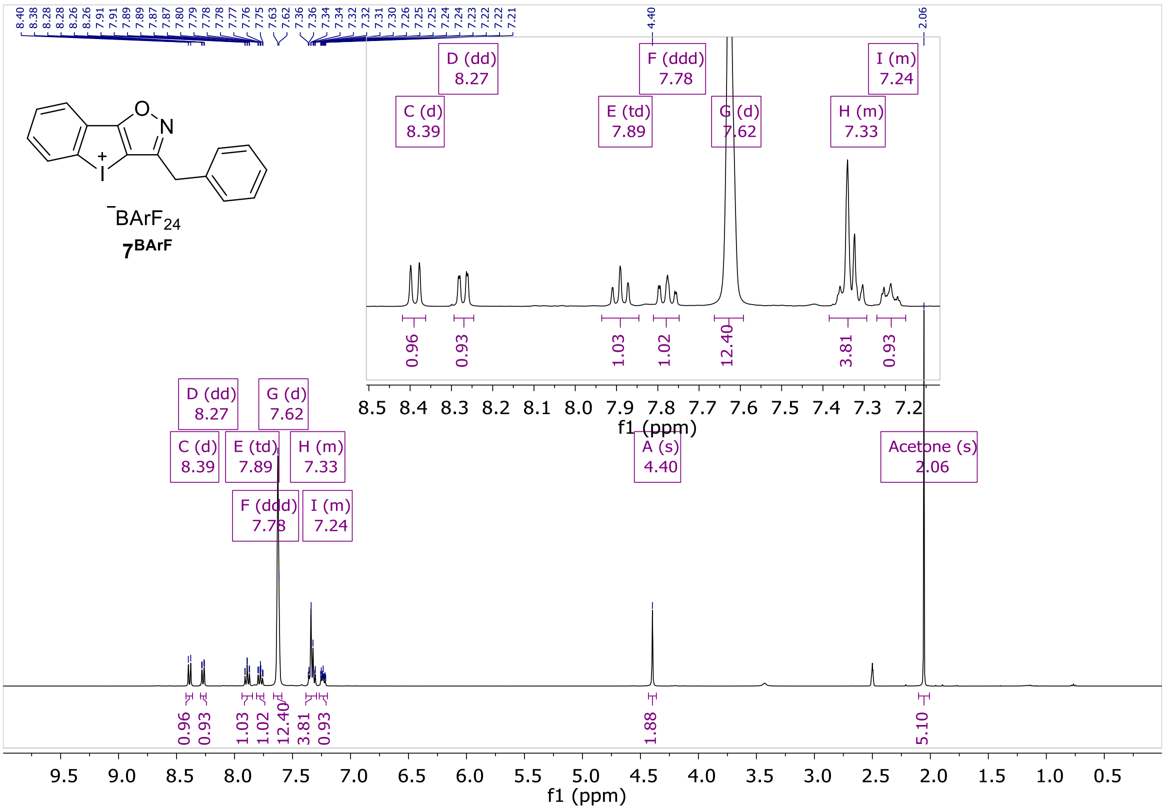

Figure S27: ^{1}H NMR spectrum of iodoloisoxazolium $\mathbf{7^{BArF}}$ in DMSO-d_6.

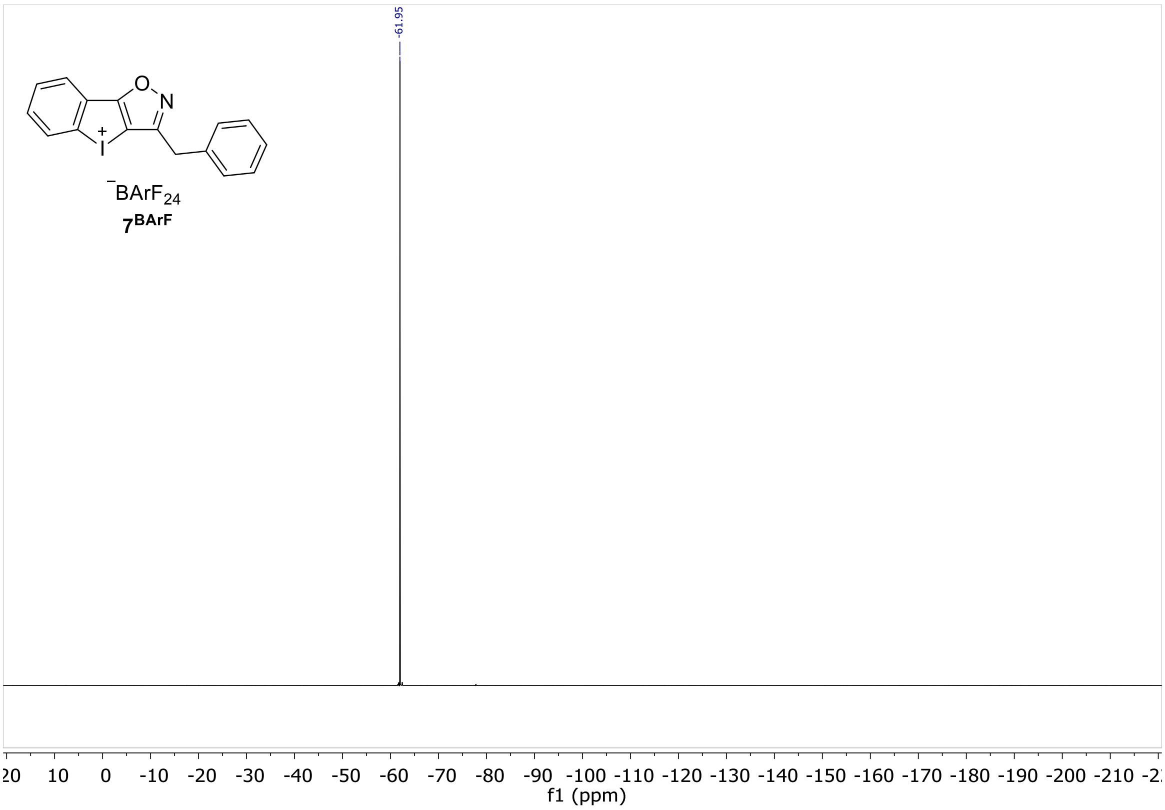

Figure S28: ^{19}F NMR spectrum of iodoloisoxazolium $\mathbf{7^{BArF}}$ in DMSO-d_6.

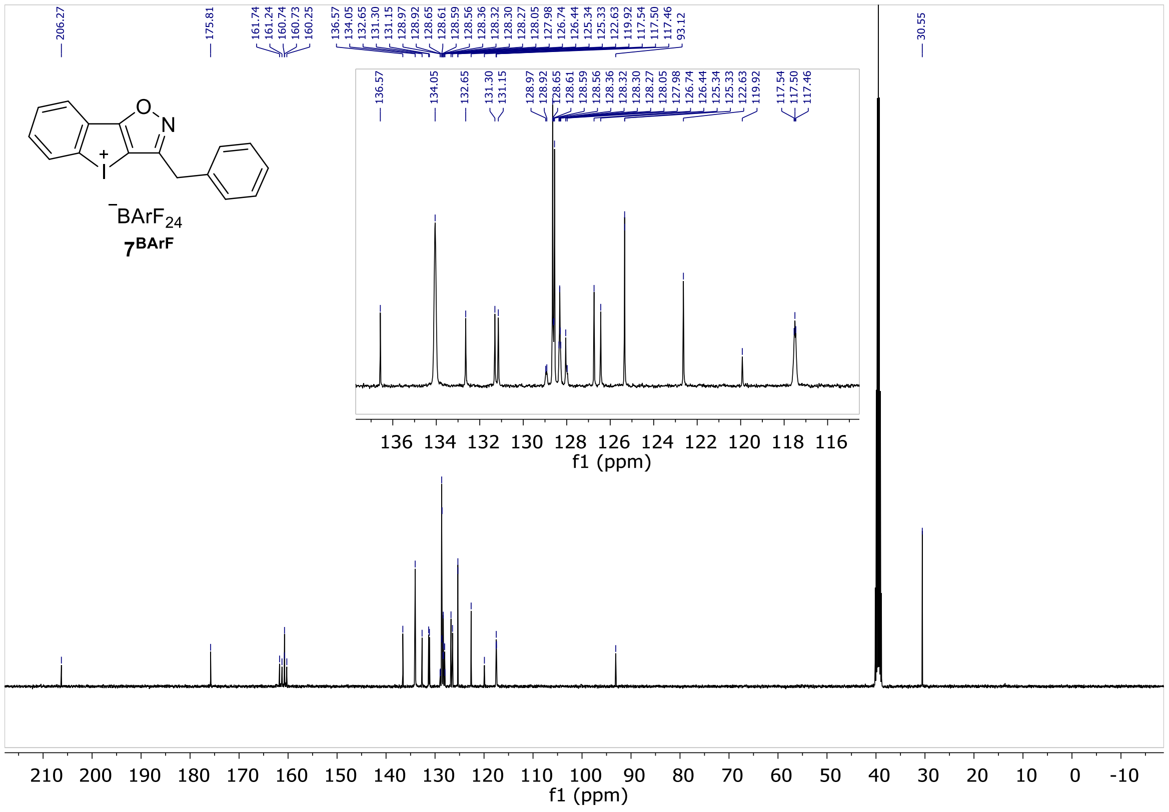

Figure S29: $^{13}C\{^{1}H\}$ NMR spectrum of iodoloisoxazolium **7^{BArF}** in DMSO-d_6.

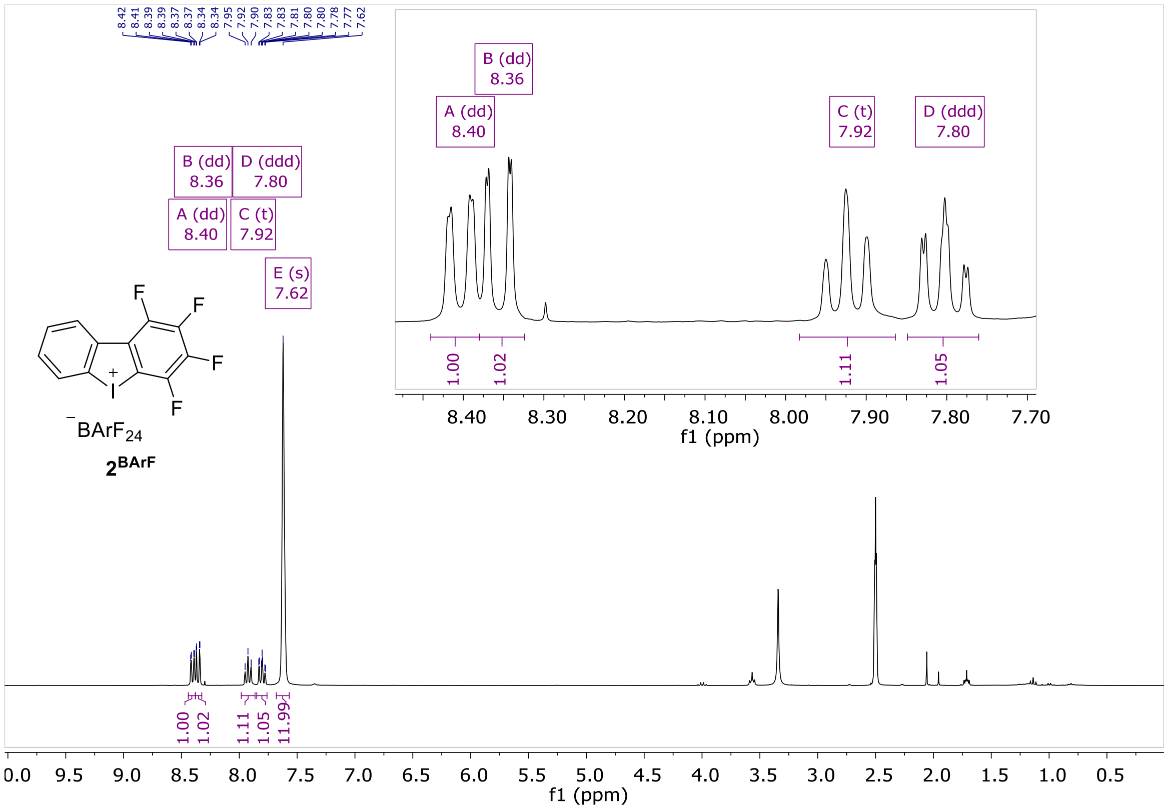

Figure S30: ^{1}H NMR spectrum of tetrafluoroiodolium **2^{BArF}** in DMSO-d_6.

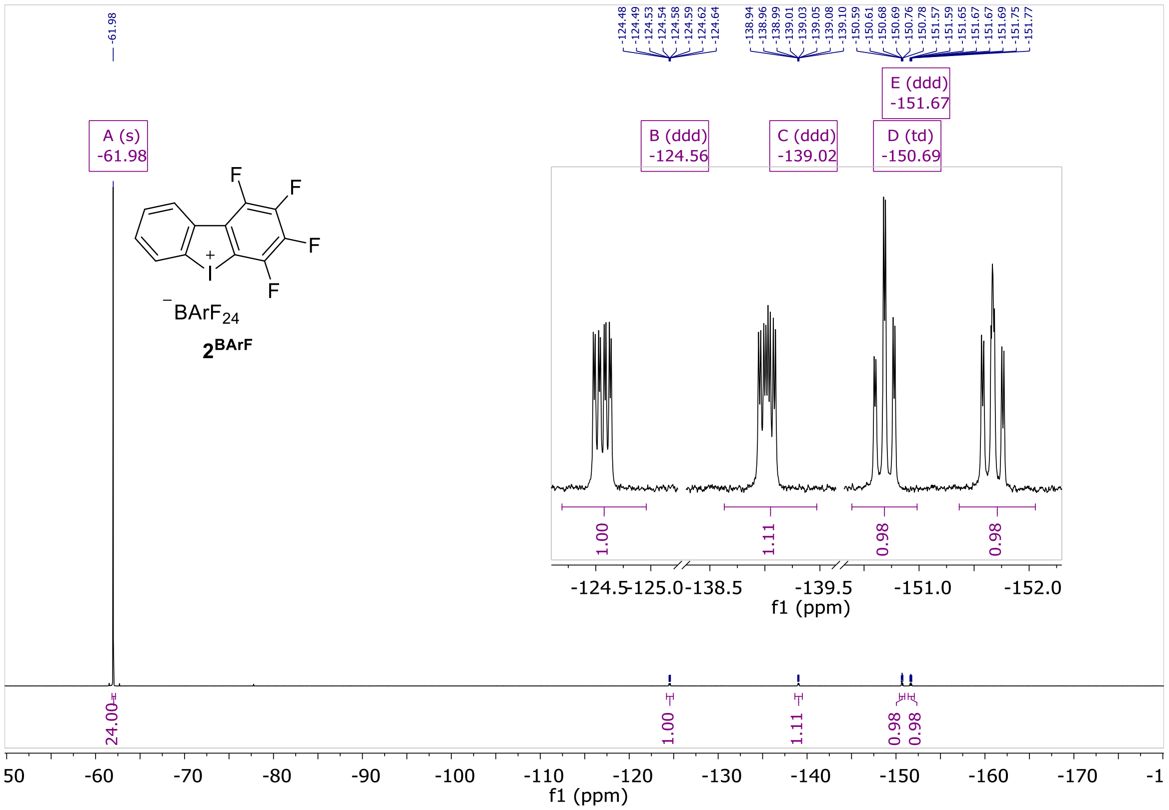

Figure S31: $^{19}F\{^{1}H\}$ NMR spectrum of tetrafluoroiodolium $\mathbf{2^{BArF}}$ in DMSO-d_6.

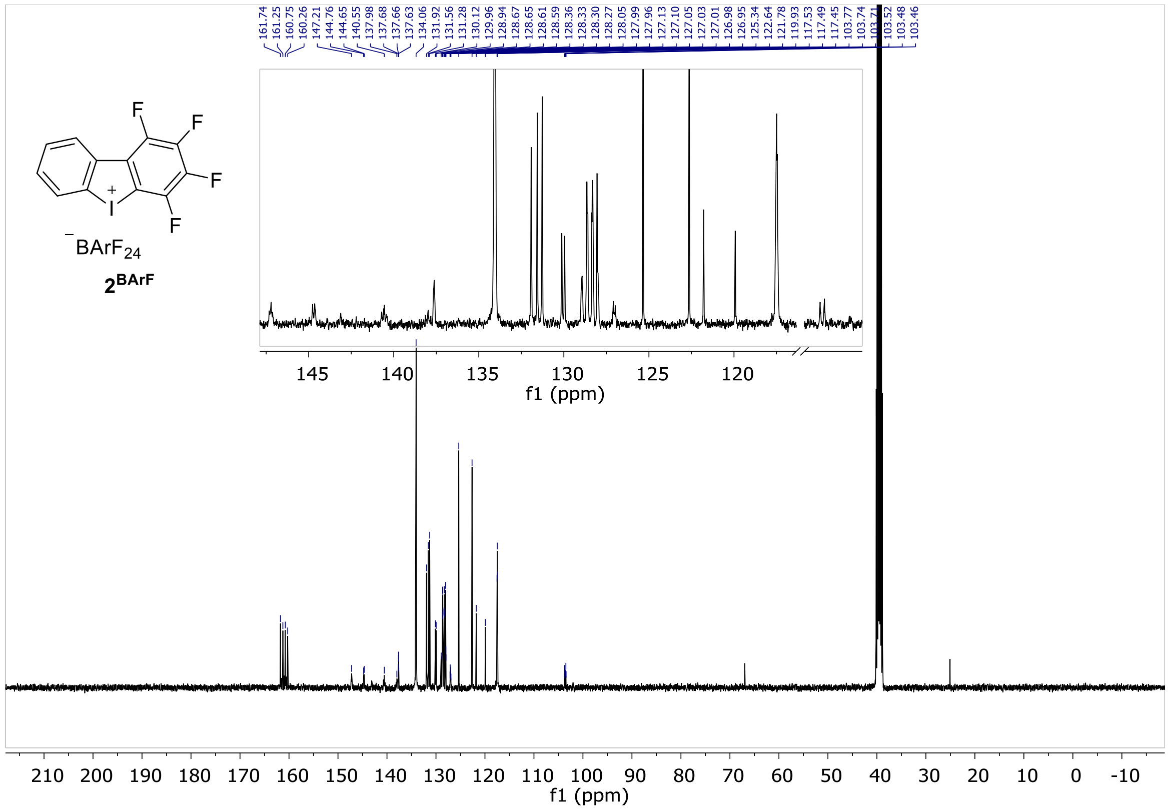

Figure S32: $^{13}C\{^{1}H\}$ NMR spectrum of tetrafluoroiodolium $\mathbf{2^{BArF}}$ in DMSO-d_6.

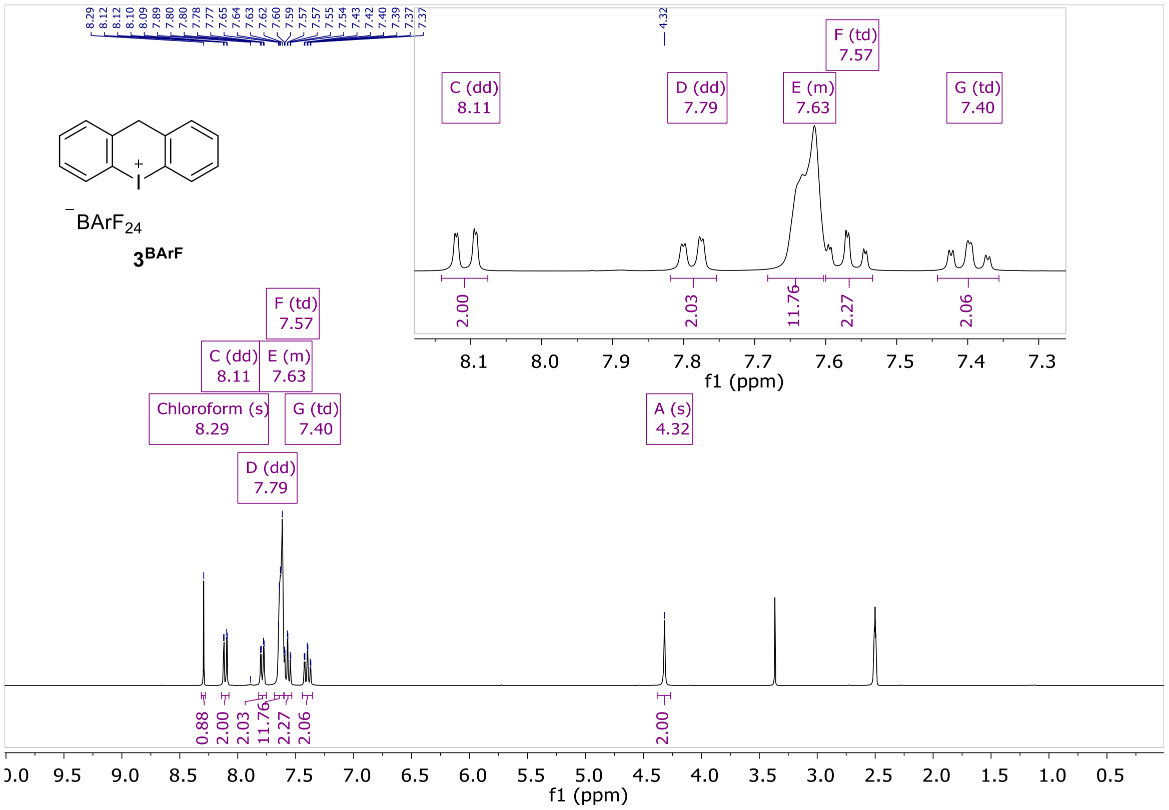

Figure S33: ^{1}H NMR spectrum of iodininium $\mathbf{3^{BArF}}$ in DMSO-d_6.

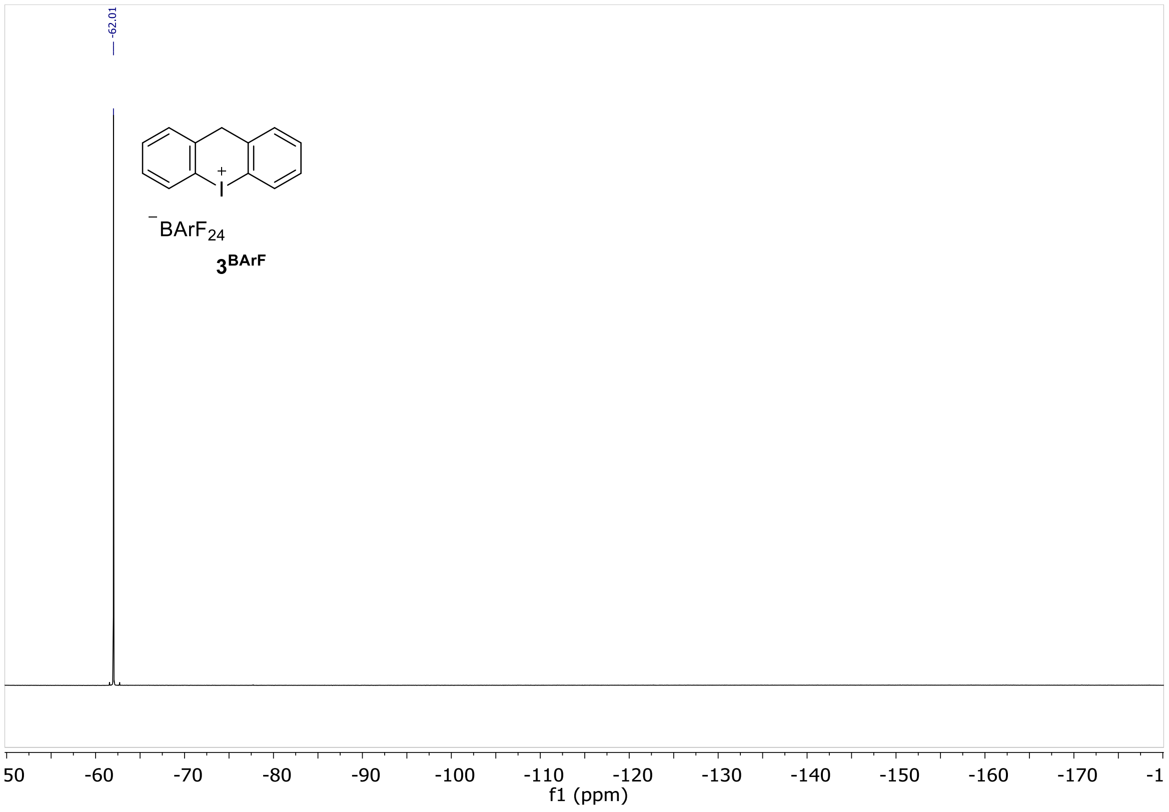

Figure S34: ^{19}F{^{1}H} NMR spectrum of iodininium $\mathbf{3^{BArF}}$ in DMSO-d_6.

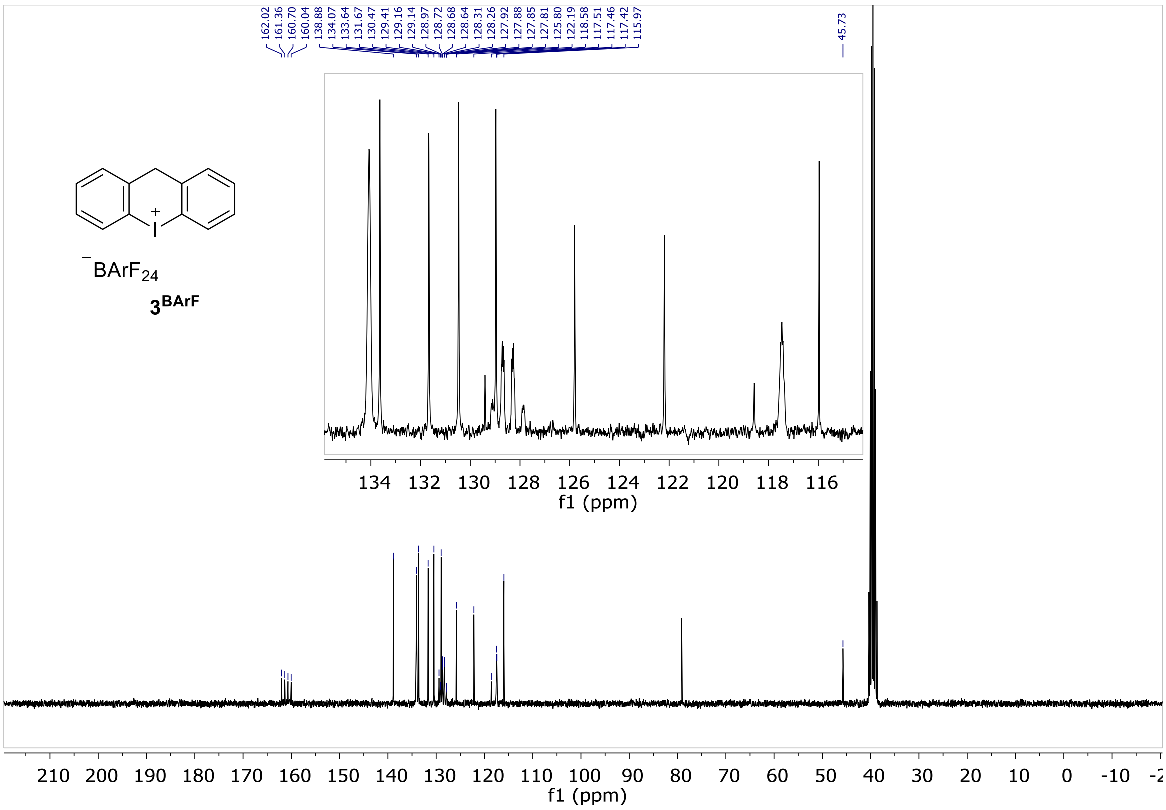

Figure S35: $^{13}C\{^{1}H\}$ NMR spectrum of iodininium $\mathbf{3^{BArF}}$ in DMSO-d_6.

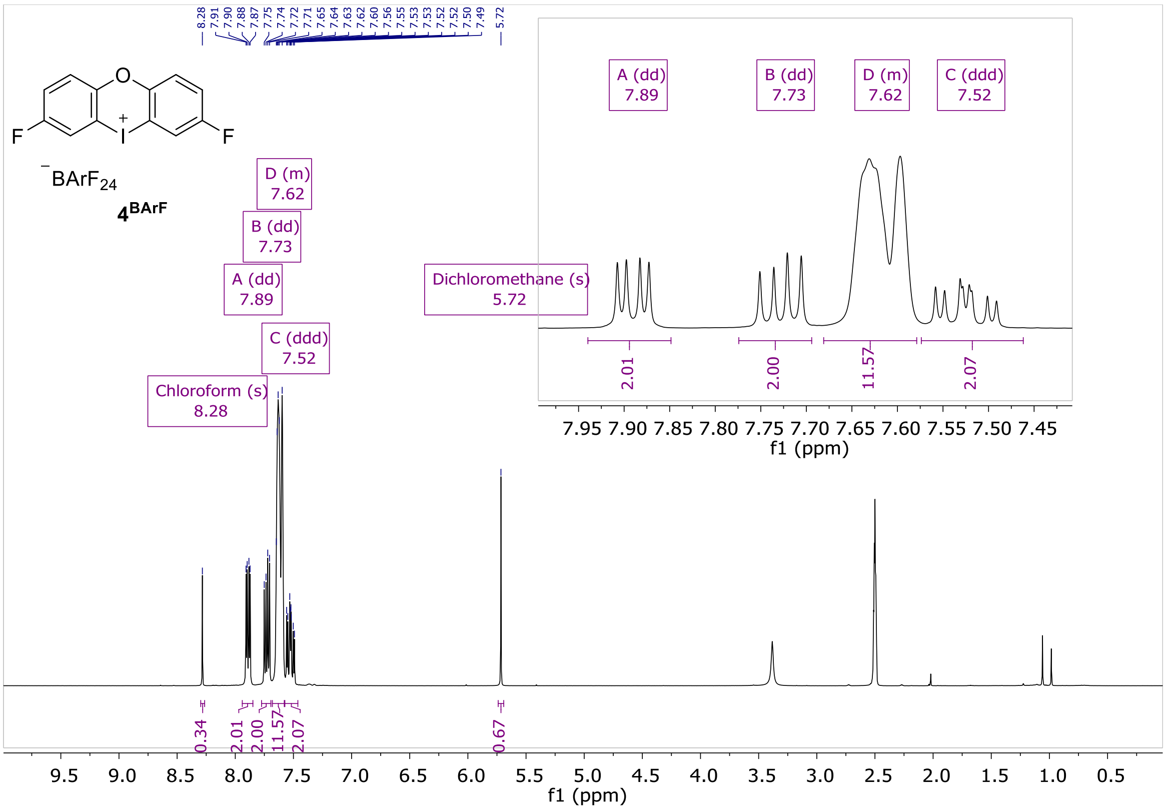

Figure S36: ^{1}H NMR spectrum of iodoxinium $\mathbf{4^{BArF}}$ in DMSO-d_6.

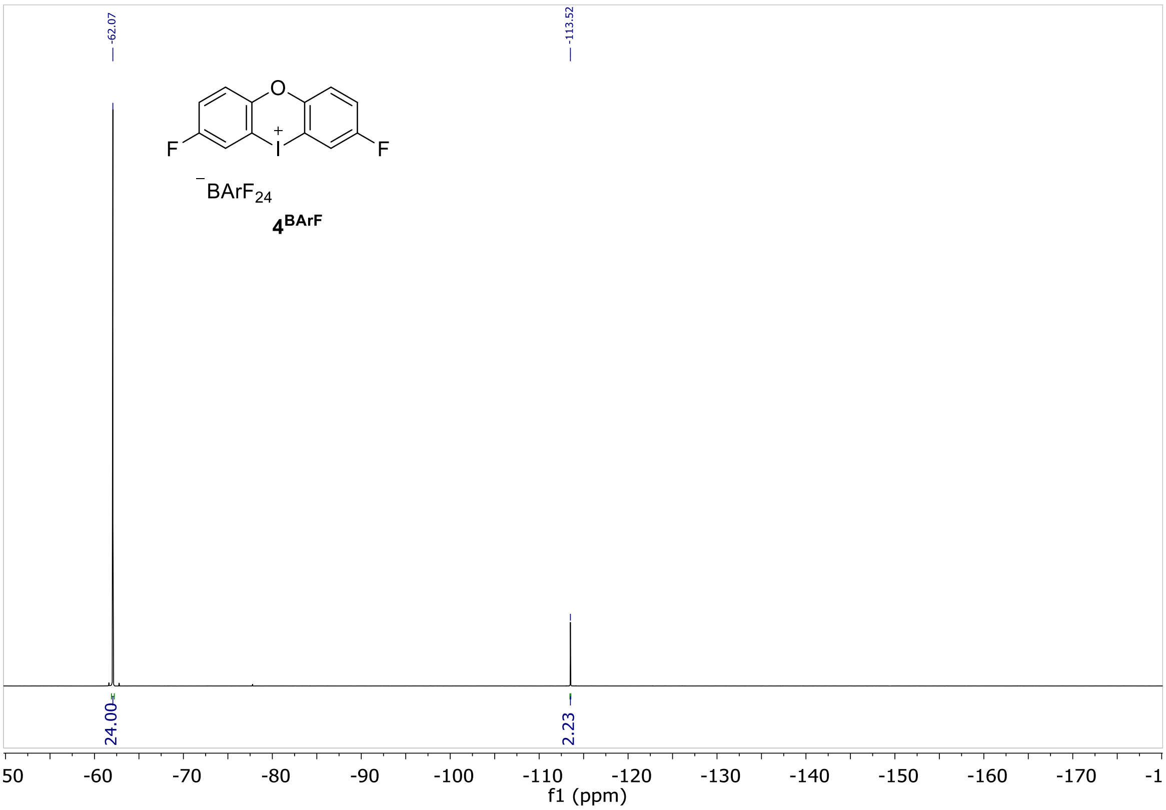

Figure S37: $^{19}F\{^{1}H\}$ NMR spectrum of iodoxinium $\mathbf{4^{BArF}}$ in DMSO-d_6.

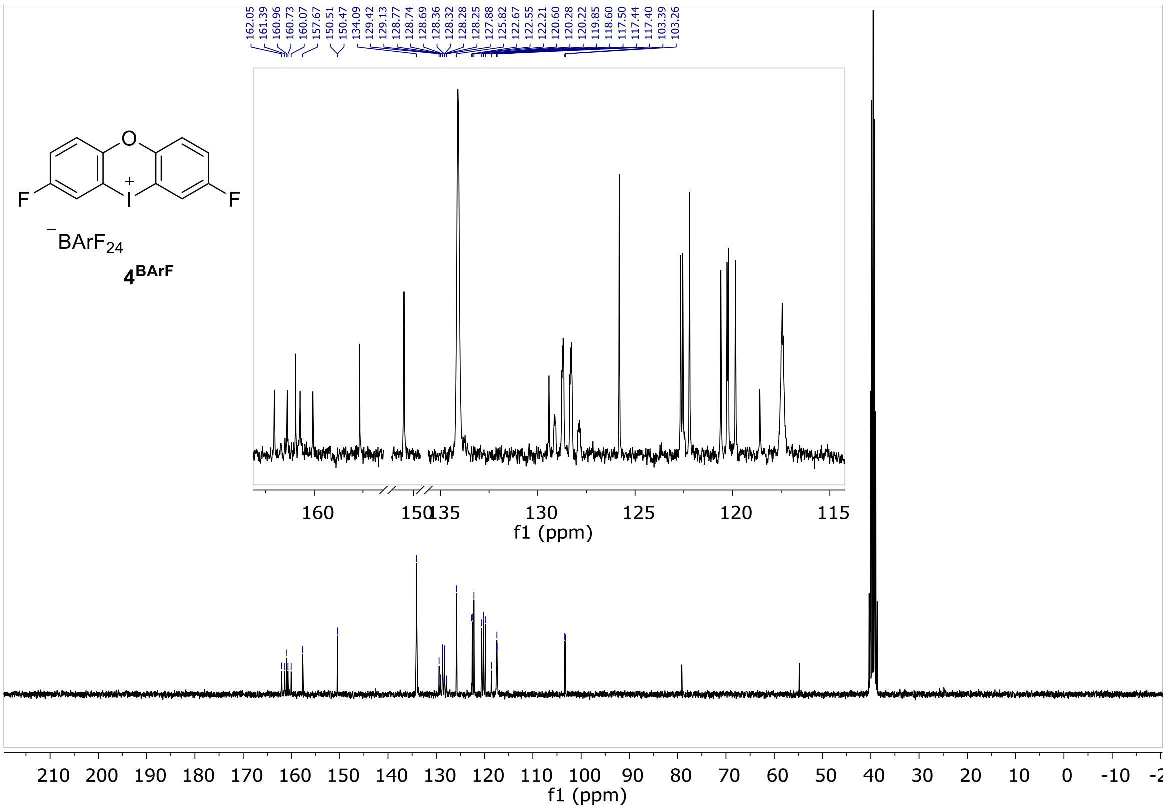

Figure S38: $^{13}C\{^{1}H\}$ NMR spectrum of iodoxinium $\mathbf{4^{BArF}}$ in DMSO-d_6.
