# Supplementary material for: Evaluating the halogen bonding strength of a iodoloisoxazolium(III) salt
Source: Beilstein J Org Chem. 2024 Sep 23;20:2401–7. doi: 10.3762/bjoc.20.204 (PMC11443664; doi:10.3762/bjoc.20.204)
Supplement: File 4 — Crystallographic data. [file Beilstein_J_Org_Chem-20-2401-s004.zip › 2362114_oc1h-dlr-cat-012-c-data_oc1h-dlr-cat-012-c_file002.html]

checkCIF/PLATON report


```
No syntax errors found.                               CIF dictionary  
Please wait while processing ....                     Interpreting this report
```

**Datablock: oc1h-dlr-cat-012-c**


---

|  |  |  |
| --- | --- | --- |
| Bond precision: | C-C = 0.0054 A | Wavelength=1.54184 |

|  |  |  |  |
| --- | --- | --- | --- |
| Cell: | a=5.4707(1) | b=10.9139(1) | c=24.3668(3) |
|  | alpha=90 | beta=95.601(1) | gamma=90 |
| Temperature: | 170 K |  |  |

|  |  |  |
| --- | --- | --- |
|  | Calculated | Reported |
| Volume | 1447.92(3) | 1447.91(3) |
| Space group | P 21/n | P 21/n |
| Hall group | -P 2yn | -P 2yn |
| Moiety formula | C32 H22 Br2 I2 N2 O2 | ? |
| Sum formula | C32 H22 Br2 I2 N2 O2 | C16 H11 Br I N O |
| Mr | 880.12 | 440.07 |
| Dx,g cm-3 | 2.019 | 2.019 |
| Z | 2 | 4 |
| Mu (mm-1) | 20.560 | 20.560 |
| F000 | 840.0 | 840.0 |
| F000' | 838.03 |  |
| h,k,lmax | 6,12,28 | 6,12,28 |
| Nref | 2560 | 2560 |
| Tmin,Tmax | 0.095,0.274 | 0.186,0.589 |
| Tmin' | 0.015 |  |

|  |  |
| --- | --- |
| Correction method= # Reported T Limits: Tmin=0.186 Tmax=0.589 AbsCorr = GAUSSIAN |  |

|  |  |
| --- | --- |
| Data completeness= 1.000 | Theta(max)= 66.482 |

|  |  |
| --- | --- |
| R(reflections)= 0.0267( 2434) | wR2(reflections)= 0.0738( 2560) |
| |  |  | | --- | --- | | S = 1.048 | Npar= 181 | |

---

```
The following ALERTS were generated. Each ALERT has the format
       test-name_ALERT_alert-type_alert-level.
Click on the hyperlinks for more details of the test.


---

Alert level B
PLAT230_ALERT_2_B Hirshfeld Test Diff for    I1       --Br1      .       12.3 s.u.  
PLAT230_ALERT_2_B Hirshfeld Test Diff for    I1       --Br1_a    .       15.0 s.u.  


---

Alert level C
PLAT934_ALERT_3_C Number of (Iobs-Icalc)/Sigma(W) > 10 Outliers ..          1 Check 
               -3  0 17,                                                        


---

Alert level G
PLAT045_ALERT_1_G Calculated and Reported Z Differ by a Factor ...      0.500 Check 
PLAT142_ALERT_4_G s.u. on b - Axis Small or Missing ..............    0.00010 Ang.  
PLAT395_ALERT_2_G Deviating  X-O-Y   Angle From 120 for O1       .      107.9 Degree
PLAT899_ALERT_4_G SHELXL2018 is Deprecated and Succeeded by SHELXL     2019/3 Note  
PLAT909_ALERT_3_G Percentage of I>2sig(I) Data at Theta(Max) Still        94% Note  
PLAT967_ALERT_5_G Note: Two-Theta Cutoff Value in Embedded .res ..      133.0 Degree
PLAT969_ALERT_5_G The 'Henn et al.' R-Factor-gap value ...........      1.967 Note  
              Predicted wR2: Based on SigI**2  3.75 or SHELX Weight  7.04       
PLAT978_ALERT_2_G Number C-C Bonds with Positive Residual Density.          2 Info  


---

   0 ALERT level A = Most likely a serious problem - resolve or explain
   2 ALERT level B = A potentially serious problem, consider carefully
   1 ALERT level C = Check. Ensure it is not caused by an omission or oversight
   8 ALERT level G = General information/check it is not something unexpected

   1 ALERT type 1 CIF construction/syntax error, inconsistent or missing data
   4 ALERT type 2 Indicator that the structure model may be wrong or deficient
   2 ALERT type 3 Indicator that the structure quality may be low
   2 ALERT type 4 Improvement, methodology, query or suggestion
   2 ALERT type 5 Informative message, check
```

---

---

It is advisable to attempt to resolve as many as possible of the alerts in all categories. Often the minor alerts point to easily fixed oversights, errors and omissions in your CIF or refinement strategy, so attention to these fine details can be worthwhile. In order to resolve some of the more serious problems it may be necessary to carry out additional measurements or structure refinements. However, the purpose of your study may justify the reported deviations and the more serious of these should normally be commented upon in the discussion or experimental section of a paper or in the "special\_details" fields of the CIF. checkCIF was carefully designed to identify outliers and unusual parameters, but every test has its limitations and alerts that are not important in a particular case may appear. Conversely, the absence of alerts does not guarantee there are no aspects of the results needing attention. It is up to the individual to critically assess their own results and, if necessary, seek expert advice. **Publication of your CIF in IUCr journals** A basic structural check has been run on your CIF. These basic checks will be run on all CIFs submitted for publication in IUCr journals (*Acta Crystallographica*, *Journal of Applied Crystallography*, *Journal of Synchrotron Radiation*); however, if you intend to submit to *Acta Crystallographica Section C* or *E* or *IUCrData*, you should make sure that full publication checks are run on the final version of your CIF prior to submission. **Publication of your CIF in other journals** Please refer to the *Notes for Authors* of the relevant journal for any special instructions relating to CIF submission. |

---

**PLATON version of 13/05/2024; check.def file version of 04/05/2024**

|  |
| --- |
| **Datablock oc1h-dlr-cat-012-c** - ellipsoid plot |
|  |

---

 Download CIF editor (publCIF) from the IUCr   
 Download CIF editor (enCIFer) from the CCDC   
 Test a new CIF entry 
